# TAPT1—at the crossroads of extracellular matrix and signaling in Osteogenesis imperfecta

**DOI:** 10.15252/emmm.202317528

**Published:** 2023-06-09

**Authors:** Julia Etich, Oliver Semler, Nicola L Stevenson, Alice Stephan, Roberta Besio, Nadia Garibaldi, Nadine Reintjes, Claudia Dafinger, Max Christoph Liebau, Ulrich Baumann, Matthias Mörgelin, Antonella Forlino, David J Stephens, Christian Netzer, Frank Zaucke, Mirko Rehberg

**Affiliations:** ^1^ Dr. Rolf M. Schwiete Research Unit for Osteoarthritis, Department of Orthopedics (Friedrichsheim) University Hospital Frankfurt, Goethe University Frankfurt/Main Frankfurt Germany; ^2^ Department of Pediatrics and Adolescent Medicine, Faculty of Medicine and University Hospital Cologne University of Cologne Cologne Germany; ^3^ Center for Rare Diseases University Hospital Cologne, University of Cologne Cologne Germany; ^4^ Center for Family Health Faculty of Medicine and University Hospital Cologne, University of Cologne Cologne Germany; ^5^ Cell Biology Laboratories, School of Biochemistry, Faculty of Life Sciences University Walk, University of Bristol Bristol UK; ^6^ Biochemistry Unit, Department of Molecular Medicine University of Pavia Pavia Italy; ^7^ Department of Biomedical Engineering The City College of New York New York NY USA; ^8^ Institute of Human Genetics, Faculty of Medicine and University Hospital Cologne University of Cologne Cologne Germany; ^9^ Department II of Internal Medicine, Faculty of Medicine and University Hospital Cologne University of Cologne Cologne Germany; ^10^ Center for Molecular Medicine Faculty of Medicine and University Hospital Cologne, University of Cologne Cologne Germany; ^11^ Institute of Biochemistry University of Cologne Cologne Germany; ^12^ Colzyx AB Lund Sweden

**Keywords:** Genetics, Gene Therapy & Genetic Disease, Musculoskeletal System

## Abstract

Osteogenesis imperfecta (OI) is a hereditary skeletal disorder primarily affecting collagen type I structure and function, causing bone fragility and occasionally versatile extraskeletal symptoms. This study expands the spectrum of OI‐causing TAPT1 mutations and links extracellular matrix changes to signaling regulation.

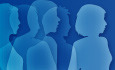

Osteogenesis imperfecta (OI) is a rare, hereditary connective tissue disorder, clinically characterized by bone fragility, bone deformities, and small stature that can be accompanied by variable extraskeletal symptoms. At the molecular level, OI is caused by a reduced quality and/or quantity of bone matrix. So far, 22 OI types are listed in the Online Mendelian Inheritance in Man (OMIM) database, and even though most of the encoded proteins have been associated with collagen type I structure and/or function, the underlying molecular mechanisms are rarely completely understood (OMIM, phenotypic series #166200). In a previous issue of this journal, a deep intronic variant in *TAPT1* was reported to create a protein‐null allele and segregated with recessive features of OI and neonatal progeria syndrome (Nabavizadeh *et al*, 2023). Using integrated analysis of RNA‐ and SI‐NET‐seq data, the authors revealed that extracellular matrix (ECM) organization and collagen‐related pathways were highly dysregulated, supporting a role for TAPT1 in ECM and collagen dynamics. However, a specific function of the TAPT1 protein remains to be identified, and also the link to collagen type I remains elusive.

In this correspondence, we provide further evidence based on our clinical, genetic, and cellular data to corroborate the role of *TAPT1* as a novel OI‐causing gene and highlight a novel disease‐relevant link between OI, ECM, and signaling. Using gene panel sequencing, we identified the homozygous mutation (c.323T>G, p.Leu108Trp) in *TAPT1* in a consanguine family (Fig 1A, upper panel). In an earlier study, mutations in *TAPT1* were reported to result in a complex and early lethal osteochondrodysplasia disrupting ciliogenesis in patient cells and leading to an altered Golgi morphology and delayed collagen secretion (Symoens *et al*, 2015). Our patient, an 18‐year‐old woman at the time of manuscript submission, was clinically diagnosed with OI type III prior to the identification of the disease‐causing gene. At the age of five (Fig 1A, middle panel), she had all typical features of classical OI, including multiple fractures, short stature (86 cm, −5.26 SDS), long bone deformities, reduced bone mineral density (0.193 g/cm^2^, −7.0 SDS, TBLH aBMD), progressive popcorn calcifications and progressive, and severe scoliosis (Fig 1A, lower panel). Remarkably, modeling of the identified mutation into the predicted 3D protein structure of TAPT1 indicated that a central network of salt bridges and hydrogen bonds between the amino acids Glu285, Asp306, Asp353, Lys356, His357, and Tyr371 on different surrounding helices is most likely being disrupted by the point mutation (c.323T>G, p.Leu108Trp), and a severe perturbation of the structural integrity is highly expected (Fig 1B upper panel). None of the tryptophan rotamers can be fitted at this position without severe clashes (Fig 1B lower panel). Prediction of the protein stability change upon mutation by multiple Cutoff Scanning Matrix method yielded an estimated loss of 2.1 kcal/mol in energy and the mutation is classified as “highly destabilizing.”

**Figure 1:**
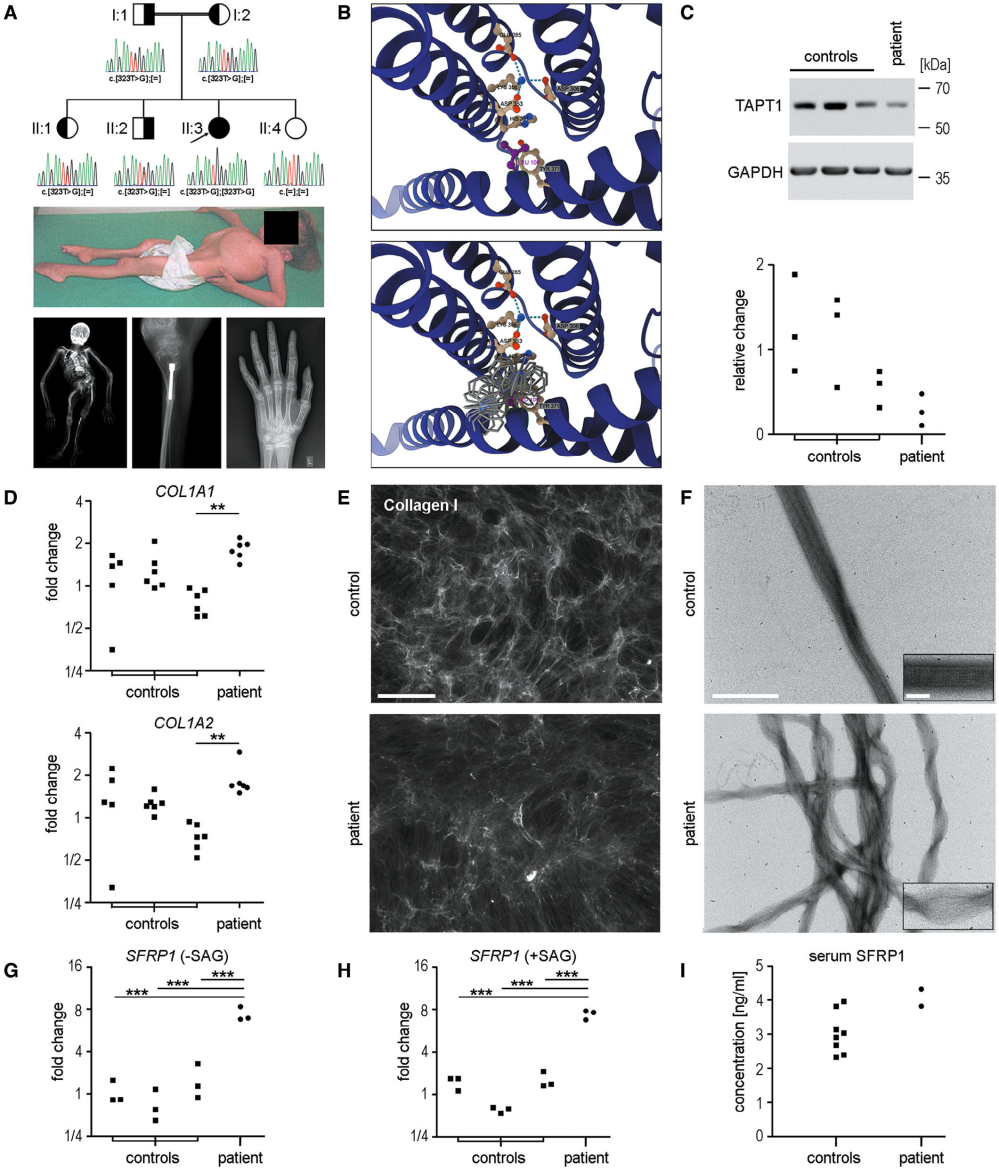
Consequences of p.Leu108Trp mutation in *TAPT1* on disease phenotype and the underlying pathomechanism (A) Pedigree of the family with the affected individual (black symbol) carrying a homozygous *TAPT1* mutation. Heterozygous carriers (half‐shaded symbols) and consanguineous relationship (double line) are highlighted (upper panel). In the affected individual (II:3) with the clinical phenotype of OI (middle panel), dual‐energy X‐ray absorptiometry (DEXA) was used to assess areal bone mineral density (aBMD) and radiographs of tibia and hand/wrist show long bones with thin cortices (bone health index 3.04, −5.68 SDS) as well as disorganized calcifications around the growth plate (also called popcorn calcifications, lower panel). (B) Model for human TAPT1 protein and the c.323 T > G, p.Leu108Trp mutation were visualized employing ChimeraX and the AlphaFold 2 model of human TAPT1 (UniProt entry Q6NXT6). Leu108 is located within an N‐terminal alpha helix protruding into the membrane bilayer and projects into a cavity surrounded by several transmembrane helices (upper panel). Glu285, Asp306, Asp353, Lys356, His357 and Tyr371 are located on several helices and are engaged in salt bridges or connected by hydrogen bonds. *In‐silico* substitution of Leu108 by a tryptophane reveals severe clashes for all rotamers (lower panel). (C) Immunoblotting of TAPT1 protein in control and patient fibroblasts (upper panel) and quantification of band intensities by ImageJ analysis (lower panel) revealed slightly reduced TAPT1 levels in patient cells. Fold change difference relative to the mean of controls is plotted as individual values from n = 3 independent experiments. Statistical analysis: One way ANOVA; *P*‐value = 0.06; (D) qPCR analysis of relative gene expression of the collagen genes *COL1A1* (upper panel) and *COL1A2* (lower panel) in control and patient fibroblasts confirmed that expression was not downregulated in patient cells. Gene expression was normalized to *GAPDH*, calibrated to the mean of controls and fold changes are plotted on a logarithmic scale form at least n = 5 independent experiments. Statistical analysis: One‐way ANOVA with *post hoc* Bonferroni multiple comparison of patient to controls a, b or c; *P*‐value [*COL1A1*] = 0.004, **c; *P*‐value [*COL1A2*] = 0.007, **c. (E) Immunofluorescence analysis of collagen type I in control (upper panel) and patient fibroblasts (lower panel) detected reduced collagen network in patient cells. Representative pictures are shown from at least n = 3 independent experiments with three control cell populations each. Scale bar: 500 μm. (F) Negative staining and transmission electron microscopy of cell culture media from control and patient cells visualized impaired collagen fibril formation in patient cells. Higher magnifications of fibers are shown as inserts. Scale bars: 500 nm (overviews), 100 nm (inserts). (G, H) qPCR analysis of relative gene expression of *SFRP1* in control and patient fibroblasts without stimulation (‐SAG, G) or upon stimulation with 1 μM smoothened agonist (+SAG, H) for 24 h hours uncovered increased *SFRP1* expression in patient cells under both conditions. Fold changes plotted on a logarithmic scale from n = 6 independent experiments are shown. Gene expression was normalized to *GAPDH* and calibrated to the mean of nonstimulated control cells from (G). Statistical analysis: One‐way ANOVA with *post hoc* Bonferroni multiple comparison of patient to controls a, b or c; *P*‐value [‐SAG] = 0.0001, ***a, ***b, ***c; *P*‐value [+SAG] < 0.0001, ***a, ***b, ***c. (I) ELISA of SFRP1 protein levels in the serum of eight healthy controls or two independent serum samples of the patient determined increased SFRP1 levels in patient serum. No statistical analysis could be performed on this dataset due to only two available patient values. Source data are available online for this figure.

To elucidate the underlying molecular pathomechanism, we used patient‐derived fibroblasts. Informed consent was obtained from the parents and the underage patient and the experiments conformed to the principles set out in the WMA Declaration of Helsinki and the Department of Health and Human Services Belmont Report. We detected *in vitro* that the p.Leu108Trp variant of TAPT1 slightly reduced protein (Fig 1C) but not RNA levels (Appendix Fig S1A). Inhibition of the proteasomal degradation machinery with the inhibitor MG132 restored TAPT1 protein levels to the levels of control cells while TAPT1 levels remained reduced in patient cells when lysosomal degradation was inhibited by Bafilomycin A1 (Appendix Fig S1B). These results indicate that mutant TAPT1 protein is mostly degraded by the proteasome pathway and are in agreement with the *in silico* analysis of mutant protein structure. We tested whether the mutation‐driven decrease in TAPT1 protein abundance will induce changes in collagen type I secretion and deposition generally associated with OI. Although we did not detect downregulated gene expression of either collagen genes, *COL1A1* and *COL1A2* (Fig 1D), we observed reduction in collagen deposition by immunofluorescence staining (Fig 1E). A slight delay of collagen secretion in patient cells was corroborated by pulse‐chase secretion kinetics (Appendix Fig S1C) and quantification of secreted or deposited collagen in cell culture medium or cell layer under steady state conditions (Appendix Fig S1D). Importantly, when analyzing the patient's extracellular quality of collagen fibrils by electron microscopy, secreted collagen was not able to assemble into banded fibrils (Fig 1F). This impaired assembly creates most likely an unorganized collagen network, which might impair the spatial interaction and composition of the ECM in affected individuals. Nabavizadeh *et al* (2023) determined by integrated pathway enrichment analysis combining RNA‐seq and SI‐NET‐seq that collagen‐ and ECM‐related pathways were most significantly dysregulated. We now prove the ECM, and particularly collagen type I assembly, as a main target of *TAPT1* mutations. Thus, our experimental data reinforce *TAPT1*‐associated OI as a collagenopathy.

We could detect some anomalies in Golgi morphology (Appendix Fig S2) but no defects in ciliogenesis (Appendix Fig S3A–E) that were previously linked to *TAPT1* mutations (Symoens *et al*, 2015). Thus, we performed functional assays and analyzed the responsiveness of the cilia‐associated hedgehog (HH) signaling pathway in smoothened agonist (SAG)‐stimulated patient fibroblasts by target gene expression. While we could detect slightly increased induction of *GLI1* and *PTCH1* expression in patient cells (Appendix Fig S3F–G), secreted frizzled‐related protein 1 (*SFRP1*) gene expression was hardly induced upon SAG stimulation. However, it was strongly upregulated in our patient compared with control cells even without stimulation by SAG (Fig 1G and H). Secreted frizzled‐related protein 1 is a secreted glycoprotein that can suppress WNT/β‐catenin signaling to regulate osteoblast differentiation and function during endochondral bone development (He *et al*, 2006). With its amino‐terminal cysteine‐rich domain SFRP1 antagonistically sequesters WNTs interfering with their binding to the Frizzled receptor to prevent β‐catenin‐mediated gene transcription, inhibit the pathway and reduce bone formation. Deletion of the *SFRP1* gene in mice resulted in increased trabecular bone mineral density and upregulated osteoblast proliferation and differentiation by preferentially activating WNT signaling in osteoblasts (Bodine *et al*, 2004). Recently, as an attempt to use WNT pathway components as potential drug targets for treating bone diseases, it was shown that inhibition of SFRP1 led to increased total bone area in a murine calvarial organ culture assay (Bodine *et al*, 2009) and to an elevated bone synthesis activity in ovariectomy‐induced osteoporotic mice (García‐García *et al*, 2022). Interestingly, osteoblasts were previously also shown to directly inhibit osteoclastogenesis through the expression, release, and binding of SFRP1 to RANKL (Häusler *et al*, 2004). The membrane‐spanning, cilia‐associated TAPT1 protein might be involved in the transcriptional regulation of *SFRP1* gene expression. It is increasingly evident that a number of cilia‐associated proteins have a nuclear presence and potentially nuclear roles (McClure‐Begley & Klymkowsky, 2017). For example, dual roles were previously described for myocardin‐related transcription factor and serum response factor that act both as transcription factors and as primary cilia constituents impacting ciliary protein–protein interactions (Speight *et al*, 2021). It is therefore conceivable that intact TAPT1 at the centrosome/ciliary base could interact with transcription‐modulating proteins and regulate their nuclear translocation. Thus, we speculate that mutant TAPT1 protein due to reduced protein stability and diminished protein interactions lacks the ability to regulate *SFRP1* silencing and consequently *SFRP1* gene expression is induced impairing bone remodeling. Bone formation (e.g., alkaline phosphatase) and bone resorption markers (e.g., deoxypyridinoline) were on the lower end within the age‐adjusted reference range in our patient (Table 1). Moreover, low bone health index with almost normal bone length in the palm but extremely thin cortical thickness indicated that the appositional bone growth mediated by the concerted interplay of bone‐forming osteoblasts and bone‐resorbing osteoclasts might indeed be a causative factor in the pathology of our patient. The importance of WNT1 in regulating remodeling‐based bone formation, cortical thickness, and appositional bone growth has been demonstrated earlier in OI‐related models (Vollersen *et al*, 2021; Wang *et al*, 2021). Strikingly, several clinical features in our patient overlap with those of OI type XV caused by mutations in *WNT1*, such as reduced cortical bone thickness, normal longitudinal bone growth, low bone turnover, progressive popcorn calcifications, and progressive scoliosis (Pyott *et al*, 2013; Liu *et al*, 2017; Nampoothiri *et al*, 2019). It is therefore possible that the clinical bone phenotype in our patient is at least in part caused by deregulated WNT signaling even though some extraskeletal symptoms do not completely overlap. Currently, we do not understand how exactly TAPT1 regulates *SFRP1* gene expression but comparison of sera from healthy donors with two independent serum samples of our patient revealed an increase in SFRP1 protein levels (Fig 1I), which underscores the possibility of persistent WNT signaling malfunction *in vivo*. This newly elucidated pathomechanism paves the way for new therapeutic approaches like the use of osteoanabolic treatments in OI (Rauch, 2017). Moreover, increased serum levels of SFRP1 might represent a potentially new and easily accessible diagnostic marker for *TAPT1*‐related OI.

**Table 1:** Clinical parameters of the patient over a period of 12 years.

Age of patient in years	5	8	12	17
Height (cm)	86	91	101	110
Height SDS	−5.26	−6.39	−7.08	−8.72
Weight (kg)	9.0	10.3	13.1	18.8
Weight SDS	−6.92	−6.39	−7.97	−11.58
BMI (kg/m^2^)	12.2	12.4	12.7	15.5
BMI SDS	−2.69	−2.52	−3.28	−2.75
TBLH‐aBMD (g/cm^2^)	–	0.352	0.428	0.610
TBLH‐aBMD aSDS (age adjusted)	–	−4.4	−4.2	−3.9
LS‐aBMD (g/cm^2^)	0.193	Not followed up due to skoliosis		
LS‐aBMD aSDS (age adjusted)	−7.000			
BAMF (range 1–10)	–	4	4	4
GMFM88 (%)	–	46.0	49.8	44.5
Spine morphology severity classification (range 1–5)	–	5.0	–	–
Spine morphology severity Score (range 1–138)	–	78.0	–	–
Nephrocalcinosis	No	No	No	No
Alkaline phosphatase (U/l)	161 (↔)	136 (↔)	136 (↔)	136 (↔)
N‐terminal procollagen type 1 propeptide (μg/l)	152 (↔)	–	–	–
Osteocalcin (ng/ml)	–	–	–	68.2 (↑*)
Deoxypyridinolin/creatinine in urine (μg/g)	–	–	–	90 (↔)
Cross‐linked carboxy‐terminal telopeptide of type I collagen (CTX) (ng/ml)	–	–	–	0.713 (↔)
Calcium/creatinine in urine (g/g)	–	–	–	0.05 (↔)
Parathyroid hormone (ng/l)	11.0 (↓)	37.0 (↔)	35.0 (↔)	35.6 (↔)
Calcium in serum (mmol/l)	2.68 (↑)	2.38 (↔)	2.49 (↔)	2.48 (↔)
Vitamin D (μg/l)	19.2 (↓)	12.9 (↓)	41.1 (↔)	34.1 (↔)
Insulin‐like growth factor I (IGF‐I) (μg/l)	–	–	–	340 (↔)

Reference values according to local laboratory or most suitable ones in literature; age adjusted if available: alkaline phosphatase: 5 years of age: < 269 U/l, others: < 300 U/l; N‐terminal procollagen type 1 propeptide: 49.9–1,200 μg/l (age adjusted); osteocalcin (*not age adjusted, premenopausal women >20): 11–43 ng/ml; deoxypyridinolin/creatinine (urine): 65–380 μg/g (adjusted to bone age); cross‐linked carboxy‐terminal telopeptide of type I collagen (CTX): 0.146–0.818 ng/ml; calcium/creatinine (urine) in g/g: < 0.21 g/g; parathyroid hormone: 15–65 ng/l; calcium (serum): until 2020: 2.20–2.65 mmol/l, from 2021: 2.10–2.55 mmol/l; vitamin D: 30–70 μg/l; Insulin‐like growth factor I (IGF‐I): 190–429 μg/l. Abbreviations are as follows: aBMD, areal bone mineral density; aSDS, age adjusted SDS; BAMF, brief assessment of motor function score; BMI, body mass index; cm, centimeters; g, gram; GMFM88, gross motor function measure 88; kg, kilogram; LS‐aBMD, lumbar spine areal bone mineral density (L2–L4); m^2^, square meter; SDS, standard deviation score; TBLH, total body less head.

The first fetuses harboring *TAPT1* mutations were initially diagnosed with lethal features of OI (Symoens *et al*, 2015). Other patients with a combination of bone fragility and variable additional symptoms have been described since then, expanding the vast phenotypic spectrum of *TAPT1* insufficiency (Jarayseh *et al*, 2023; Nabavizadeh *et al*, 2023). The impact of the identified mutations ranges from partial to complete loss‐of‐function most likely causing the phenotypic variability from early lethal to survivable disease manifestation. The reported phenotypes associating with poor mineralization (Symoens *et al*, 2015), skeletal osteopenia (Jarayseh *et al*, 2023), and calcification defects (Nabavizadeh *et al*, 2023) are similar to those seen in our patient. In summary, we provide a first mechanistic indication that *SFRP1* gene expression is induced in patient cells harboring the c.323T>G *TAPT1* mutation and potentially other mutations affecting TAPT1 protein levels and/or stability to presumably deregulate bone remodeling. Thus, we propose that mutations in *TAPT1* can cause OI of variable severity through impaired collagen fibril formation and deregulation of bone turnover.

## Author contributions

**Julia Etich:** Conceptualization; formal analysis; supervision; validation; investigation; visualization; methodology; writing – original draft; writing – review and editing. **Jörg Oliver Semler:** Conceptualization; supervision; funding acquisition; writing – review and editing. **Nicola Stevenson:** Formal analysis; investigation; writing – review and editing. **Alice Stephan:** Formal analysis; investigation; writing – review and editing. **Roberta Besio:** Formal analysis; investigation; writing – review and editing. **Nadia Garibaldi:** Formal analysis; investigation; writing – review and editing. **Nadine Reintjes:** Formal analysis; investigation; writing – review and editing. **Claudia Dafinger:** Formal analysis; investigation; writing – review and editing. **Max Christoph Liebau:** Formal analysis; investigation; writing – review and editing. **Ulrich Baumann:** Formal analysis; investigation; writing – review and editing. **Matthias Mörgelin:** Formal analysis; investigation; writing – review and editing. **Antonella Forlino:** Formal analysis; supervision; writing – review and editing. **David John Stephens:** Formal analysis; supervision; writing – review and editing. **Christian Netzer:** Formal analysis; supervision; writing – review and editing. **Frank Zaucke:** Conceptualization; supervision; funding acquisition; writing – review and editing. **Mirko Rehberg:** Conceptualization; investigation; visualization; methodology; writing – original draft; writing – review and editing.

## Disclosure and competing interests statement

The authors declare that they have no conflict of interest.

## Supporting information



Appendix

Source Data for Figure 1
