# Supplementary material for: TAPT1—at the crossroads of extracellular matrix and signaling in Osteogenesis imperfecta
Source: EMBO Mol Med. 2023 Jun 9;15(7):e17528. doi: 10.15252/emmm.202317528 (PMC10331569; doi:10.15252/emmm.202317528)
Supplement: Supplementary file 1 — Appendix [file EMMM-15-e17528-s001.pdf]

APPENDIX

Table of contents

FIGURES 2
Appendix Figure S1 2
Appendix Figure S2 4
Appendix Figure S3 5
MATERIALS AND METHODS 7
Clinical characterization of the patient 7
Genetic characterization of the patient 8
Bioinformatic prediction of protein structure and stability upon mutation 9
Human tissues 9
RNA isolation and quantitative (q)PCR 10
Immunoblotting 11
Immunocytochemistry 12
Steady-state and pulse-chase analyses of fibrillar collagen 13
Determination of Protein Concentration 14
Electron microscopy 14
Statistical analysis 14
REFERENCES 15

A

FIGURES

Appendix Figure S1

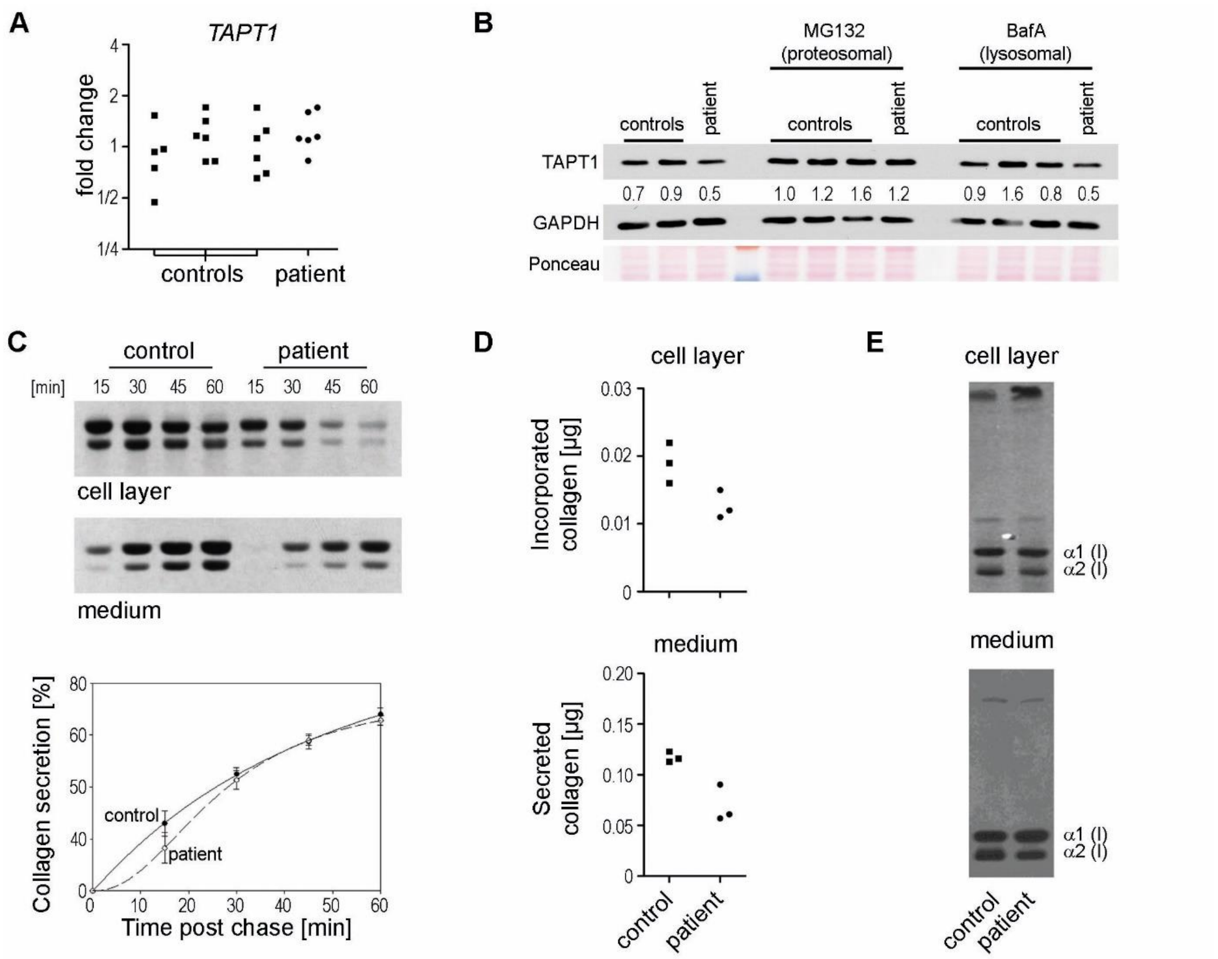

Appendix Figure S1 - Consequences for collagen expression, secretion and deposition.

A Relative gene expression of *TAPT1* in control and patient fibroblasts was assessed by qPCR. Gene expression was normalized to *GAPDH*, calibrated to the mean of controls and fold changes are plotted on a logarithmic scale from at least n = 5 independent experiments. Statistical analysis: One way ANOVA; *P*-value = 0.4*.*

B Immunoblotting of TAPT1 protein in control and patient fibroblasts cultured in the presence of MG132 (5 mM) a proteasome inhibitor or Bafilomycin A1 (100 nM), an inhibitor of autophagosome-lysosome fusion and lysosomal degradation. GAPDH and Ponceau staining were used as loading controls. Band intensities of TAPT1 were normalized to band intensities of GAPDH and calibrated to the mean of all samples and relative expression levels are indicated as numbers.

C Collagen secretion was evaluated by incubating the cells with ^{3}H-proline for 4 h followed by extraction from both cell layer and medium fractions at 15, 30, 45 and 60 min after the pulse. Samples were run on non-reducing SDS-urea-PAGE (upper panel) and the ratio between the densitometric value of collagen I in the media and the total collagen I present in medium and

in cell layer was evaluated to quantify the percentage of collagen secretion for each time point. The percentage of collagen secretion is plotted against the collection time points (lower panel).

D The amount of collagen incorporated into the cell layer (upper panel) or secreted into the cell culture medium (lower panel) was quantified in n = 3 independent experiments. Statistical analysis: Mann-Whitney-U; *P*-value [cell layer] = 0.1; *P*-value [medium] = 0.1.

E Non-reducing electrophoretic analyses of type I collagen extracted from control and patient fibroblasts. Cells were labeled with ^{3}H-proline for 18 h. Type I collagen was extracted from cell layer and medium fractions and analyzed by SDS-PAGE. Representative fluorographs are shown.

<u>Appendix Figure S2</u>

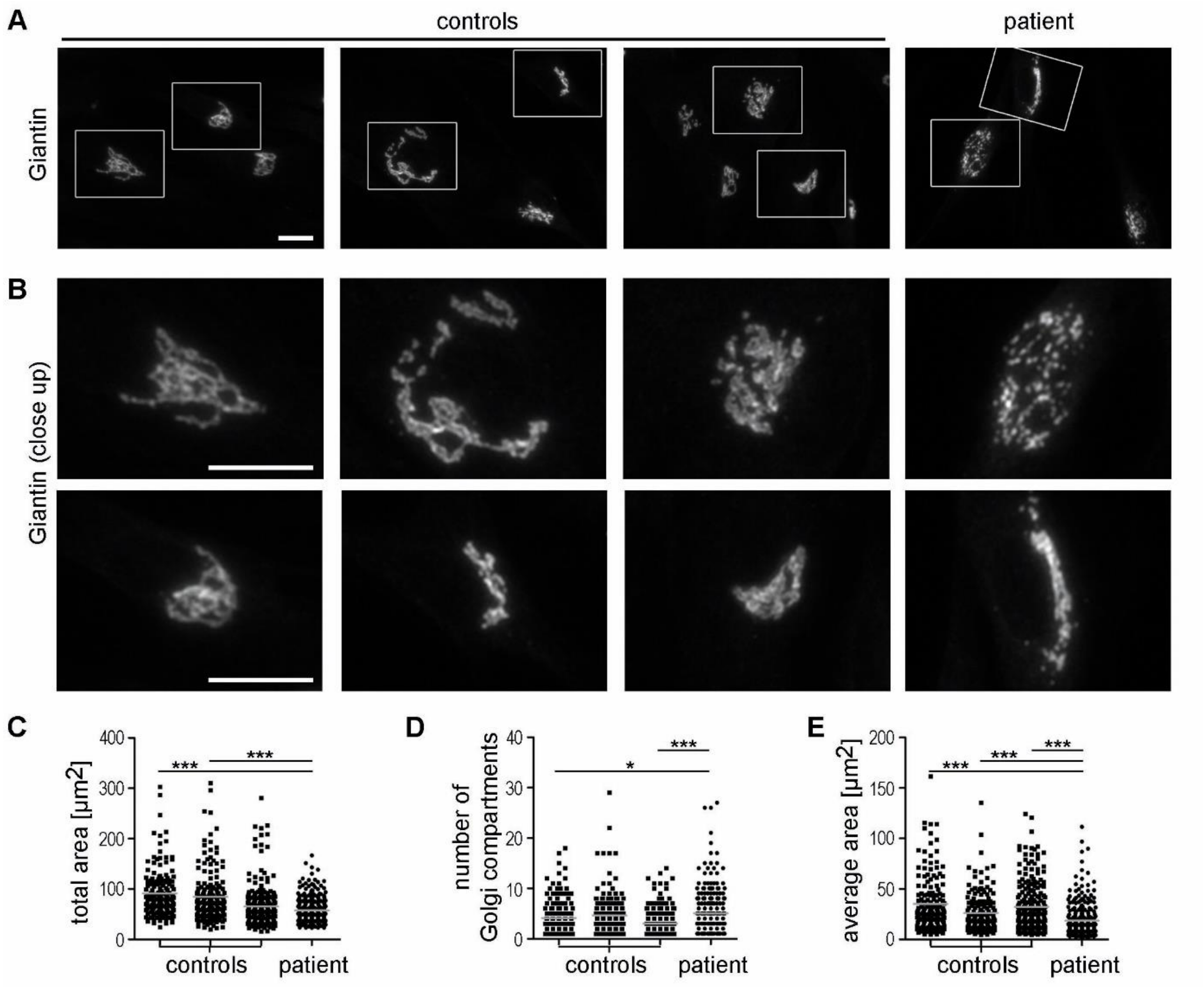

Appendix Figure S2 - Impact on Golgi morphology.

A-B Golgi apparatus in sub-confluent control and patient fibroblasts was visualized by immunofluorescent detection of giantin. Overviews are shown in (A) and zoomed areas shown in (B) are highlighted. In patient fibroblasts fragmented (B, upper panel) as well as condensed Golgi (B, lower panel) could be observed more frequently as compared to control cells. Scale bars: 20µm.

C-E Golgi total area (C), number of Golgi compartments per cell (D) and average area of Golgi compartments per cell (E) were quantified by particle analysis in ImageJ from n = 3 independent experiments. Statistical analysis: One way ANOVA with post hoc Bonferroni multiple comparison of patient to control a, b or c; *P*-value [total area] < 0.0001, ***a, ***b*; P*-value [number] < 0.0001; *a, ***c*; P*-value [average area] < 0.0001, ***a, ***b, ***c.

Appendix Figure S3

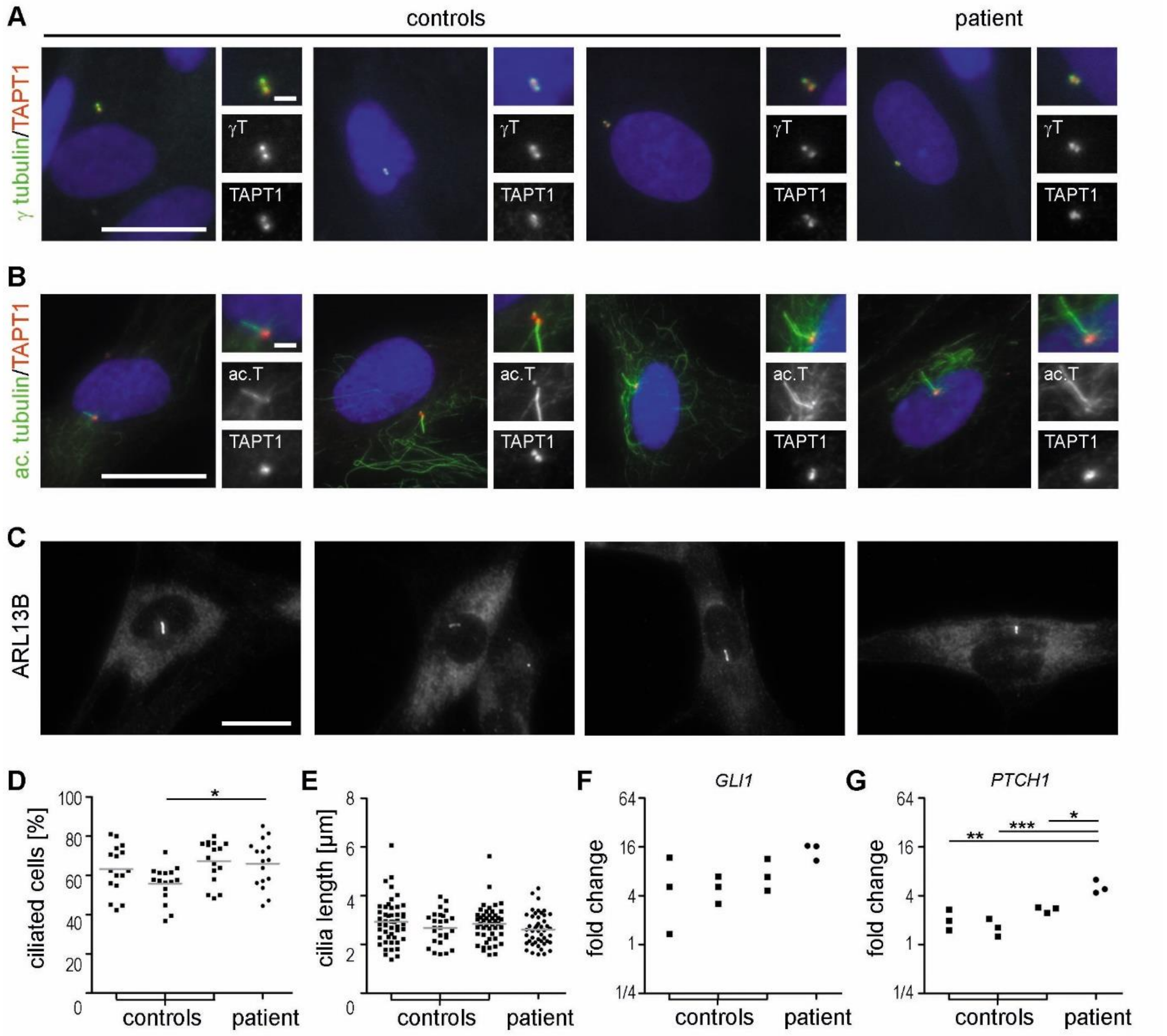

Appendix Figure S2 - Localisation of mutated TAPT1 and its influence on ciliogenesis and cilia-associated signalling pathways.

A-B Subcellular localization of TAPT1 protein was determined in control and patient cells by immunofluorescence analysis and co-labelling of the centrosome (γ tubulin, A) and cilia (acetylated (ac.) tubulin, B) after induction of cilia formation by starvation for 24 h. Scale bars: 20µm (overview), 2µm (inserts).

C Cilia were detected by immunofluorescence analysis as ARL13B-positive structures after induction of cilia formation by starvation for 24 h. Scale bar: 20µm.

D-E The proportion of ciliated cells from n = 2 independent experiments (D) and the length of cilia irrespective of orientation from n = 3 independent experiments (E) were quantified using ImageJ analysis. Statistical analysis: One way ANOVA with post hoc Bonferroni multiple comparison of patient to controls a, b or c; *P*-value [ciliated cells] = 0.024, *b*; P*-value [length] = 0.2*.*

F-G Target gene expression analysis of HH-induced *GLI1* (D) and *PTCH1* (E) was analyzed by qPCR after stimulation of control and patient fibroblasts with 1µM smoothened agonist for 24 h in n = 3 independent experiments. Gene expression was normalized to *GAPDH*,

calibrated to corresponding non-stimulated cells and fold changes are plotted on a logarithmic scale. Statistical analysis: One way ANOVA with post hoc Bonferroni multiple comparison of patient to controls a, b or c; *P*-value [*GLI1*] = 0.1715*; P*-value [*PTCH1*] = 0.0011, **a, ***b, *c*.*

MATERIALS AND METHODS

Clinical characterization of the patient

The patient was regularly characterized, monitored and treated at our outpatients department for rare skeletal dysplasias by certified paediatricians with experience in OI. Clinical follow up and laboratory tests were performed within the clinical routine of patients care with standard methods. All measuring devices were approved for medical use and calibrated according to the manufacturer's specifications. DXA scans were performed using Prodigy Advance® (2006 – 2010) or iDXA (2011 – 2021; both from GE Healthcare, Buckinghamshire, UK) with the Encore® software versions 10 to 14. To unify for algorithm version and acquisition mode, the raw data of every measurement was recalculated with the same, then most recent Encore software) on iDXA. Motoric assessments were performed by trained physiotherapists according to the published protocols, also within our routine patients care (Cintas *et al*, 2003; Ruck-Gibis *et al*, 2001). Spine morphology, severity score and classification, was assessed using a standard assessment (Koerber *et al*, 2012). Reference values were applied according to the assay used in the local laboratory at that time and/or the most suitable published set of reference values. Age specific norm-values were used if available because bone metabolism is highly dependent on growth velocity and therefore age. Skin biopsy was taken after informed consent of both parents and the patient during a routine surgery for removal of an intramedular rod. This procedure and the scientific workup were approved by the local ethics committee (AZ 13-202) and the experiments conformed to the principles set out in the WMA Declaration of Helsinki and the Department of Health and Human Services Belmont Report. A consent to publish patient information was obtained by the family.

Genetic characterization of the patient

A gene panel sequencing was performed in the patient using a custom designed Agilent SureSelect enrichment kit for 892 genes associated with skeletal disorders on an Illumina NextSeq (San Diego, CA, USA). Next-Generation-Sequencing platform at a mean sequence coverage of 439 (1x) and 98.9 % of the target region sequenced at 10x. Mapping of reads to the hg19 reference genome, variant calling, annotation and filtering was performed with the QIAGEN (Hilden, Germany) CLC Biomedical Workbench and in-house developed annotation and variant filtering software. The gene panel dataset was filtered for *de novo*, homozygous, possible compound-heterozygous and heterozygous variants (minor allele frequency (MAF) <0.5% for recessive variants and <0.01% for dominant variants in the gnomAD database) with a coverage of more ≥10 reads. Additionally, the dataset was filtered for variants with pathogenic *in silico* prediction (CADD ≥15) and variants with a previously described disease association as listed in the Human Gene Mutation Database (HGMD Professional 2018.2) or in the ClinVar archive (latest accessions in August 2019). Detected variants were classified according to the American College of Medical Genetics and Genomics (ACMG) guidelines for variant classification (Richards *et al*, 2015). In none of the genes of the panel, variants with a clear association to the skeletal phenotype were detected. Additionally, target region coverage-based copy number variant detection was performed on the NGS dataset and did not reveal any deleterious CNVs. In the context of the index patient's phenotype, only the homozygous variant c.323T>G (p.L108W) in the gene TAPT1 (NM_153365.2) remained as a possibly disease-associated variant. The variant was as "variant of unknown significance" with the ACMG criteria PM2 (absent from controls) and PP3 (deleterious *in silico* prediction). This variant was subsequently Sanger sequenced using the ABI BigDye terminator v3.1 chemistry (Thermo Fisher Scientific) on an ABI 3500 Genetic Analyzer

(Thermo Fisher Scientific) in the patient, both parents and in the three siblings and was confirmed to segregate with the phenotype in family. Since the phenotype of the index patient is somewhat different from the TAPT1-associated phenotype described so far, the ACMG PP1 criterion (cosegregation with disease in multiple affected family members in a gene definitively known to cause the disease) could not be assigned. The variant was deposited at ClinVar (Accession: VCV002500273.1, Variation ID: 2500273, https://www.ncbi.nlm.nih.gov/clinvar/variation/2500273/).

Bioinformatic prediction of protein structure and stability upon mutation

The mutation (c.323T>G, p.Leu108Trp) was visualised employing ChimeraX (Pettersen *et al*, 2021) and the AlphaFold 2 (Jumper *et al*, 2021) model of human TAPT1 (UniProt entry Q6NXT6). The pLLDT score of the model in this region is > 96, thus implying a highly reliable structure prediction. A prediction of the (c.323T>G, p.Leu108Trp) mutation on protein stability was performed employing the multiple Cutoff Scanning Matrix method (mCSM; https://biosig.lab.uq.edu.au/mcsm/) and the predicted structure of TAPT1 (Pires *et al*, 2014).

Human tissues

Human fibroblast cultures were established from skin biopsies of the female patient at the age of 15 years, grown and maintained in Dulbecco Modified Eagle's Medium (DMEM, 4.5 g/l glucose) with 10% fetal calf serum (FCS), 100 µg/ml penicillin/streptomycin at 37°C in humidified atmosphere containing 5 % CO_2. Juvenile controls were kindly provided by Christian Schaaf (Heidelberg) and comprise juvenile donors of different sex and age to cover a broad range of biological variability and minimize over-interpretation of data due to individual characteristics of the donors. To analyze cilia formation or Golgi morphology, cells were plated at $1x10^4$ cells/cm^2 on

glass for one day and starved for 23-25 h prior to immunofluorescence analysis. For gene expression analysis and collagen production, cells were plated at $4x10^4$ cells/cm^2 on plastic (RNA) or glass (immunocytochemistry) and medium was supplemented with ascorbate (22 µg/ml ascorbic acid, 65 µg/ml ascorbic acid-2-phosphate). To induce HH signaling, cells were cultured at $4x10^4$ cells/cm^2 for two days in the presence of ascorbate and incubated for further 23-25 h with 1 µM smoothened agonist (SAG, Thermo Fisher Scientific) in ascorbate-free culture medium containing 0.5 % FCS. To inhibit proteasomal or lysosomal degradation cells were cultured for 16 h in the presence of 5 mM MG132 (Sigma Aldrich) or for 4 h in the presence of 100 nM Bafilomycin A1 (Alfa Aesar), respectively.

RNA isolation and quantitative (q)PCR

Total RNA was isolated by phenol-chloroform extraction using TRIzol reagent and the concentration of RNA was determined using NanoDrop 2000 spectrophotometer. RNA was reversely transcribed into cDNA with the Omniscript RT assay and 25 ng cDNA was used for SYBR Green-based qPCR assays (Eurogentec) in a total volume of 25 µl in clear FrameStar 96 semi-skirted plates (4titude) on StepOnePlus™ Real-Time PCR System (Applied Biosystems). Thermal cycling parameters were 3 min at 95 °C followed by 40 cycles of 10 s at 95 °C, 20 s at 60 °C, 40 s at 72 °C and specificity of gene amplification was confirmed by melt curve analysis. The expression was normalized to *GAPDH*, the fold change was calculated with the ΔΔCT method (Pfaffl, 2001) , log2-transformed and displayed on a log2-scaled axis to represent the expression changes in a similar manner across the order of magnitude. The following primers were used:

COL1A1 (forward: 5′-AGATGGACTCAACGGTCTCC-3′; reverse: 5′-AGGAAGCTGAAGTCGAAACC-3′);

COL1A2 (forward: 5′-TGCTGGCAAACATGGAAACCG-3′; reverse: 5′-GCAGACCTTGCAATCCATTGTG-3′);

GAPDH (Besio *et al*, 2019) (forward: 5′-ATACCAGGAAATGAGCTTGACAAA-3′; reverse: 5′-TCCTCTGACTTCAACAGCGACAC-3′);

GLI1 (Wang *et al*, 2012) (PrimerBank ID 263190679c1; forward: 5′-AGCGTGAGCCTGAATCTGTG-3′; reverse: 5′-CAGCATGTACTGGGCTTTGAA-3′);

PTCH1 (Wang *et al*, 2012) (PrimerBank ID 134254431c3; forward: 5′-ACTTCAAGGGGTACGAGTATGT -3′; reverse: 5′-TGCGACACTCTGATGAACCAC -3′);

SFRP1 (forward: 5′-GCTTAAGTGTGACAAGTTCCC-3′; reverse: 5′-CATCCTCAGTGCAAACTCG-3′);

TAPT1 (forward: 5′-GATGGGCTTTATTCCTCTCC-3′; reverse: 5′-CATACTGGCACGATTTCCC-3′).

Immunoblotting

Trypsinized, washed cell pellets were resuspended in transmembrane protein buffer (10 mM Tris-HCl pH7.4, 150 mM NaCl, 1 % triton X-100, 1 mM β-mercaptoethanol, 1 mM EDTA pH 8.0, 1 mM iodoacetamid) containing cOmplete protease inhibitor (Roche), passed through a 27G needle and lysed for 1 h at 4 °C while end-to-end rotating. After sonification and centrifugation, clear supernatant was supplemented with sample buffer (62.5 mM Tris-HCl, pH6.8, 2 % SDS, 10 % glycerol, 0.04 % bromophenol blue, 0.5 % β-mercaptoethanol) and incubated for 10 min at room temperature. Equal amounts were resolved on SDS-polyacrylamide gels, transferred onto nitrocellulose, incubated with primary antibodies detecting TAPT1 (Merck; HPA042567; 1/4000) and GAPDH (Merck; MAB374; 1/1500), detected with corresponding secondary antibodies coupled with horseradish peroxidase (DAKO) and

visualized by chemoluminescence. For quantification, exposure was adjusted to the linear range for each of the analyzed targets. Band intensities were determined using ImageJ software, samples were normalized to GAPDH control and calibrated by the mean of controls.

Immunocytochemistry

For assessment of collagen type I or giantin, cells were fixed with ice cold 50% methanol/50% acetone. Cells were incubated with primary antibodies specific for collagen type I (Abcam; ab34710; 1/200) or giantin (Enzo; ALX-804-600-C100; 1/200) in 5% normal goat serum. For analysis of cilia length, serum starved cells (24 h) were fixed with 4% paraformaldehyde, permeabilized in 5% normal goat serum containing 0.1% triton X-100, stained with a primary antibody specific for ARL13B (Proteintech, 17711-1-AP; 1/500). Corresponding secondary antibodies labelled Cy3 (Jackson Laboratories) or Alexa-Fluor 488 (Thermo Fischer Scientific) were used to visualize the binding of the primary antibodies, nuclei were counterstained with DAPI (4,6-diamidino-2-phenylindole; Life Technologies, D1306), coverslips mounted in Mowiol and analyzed by fluorescence microscopy (NikonEclipse TE2000-U Microscope or Zeiss AxioPhot Fluorescent Microscope with Nikon digital sight DS-2MV camera). For subcellular localization of TAPT1 protein serum starved cells (24 h) were fixed in methanol for 3 min at -20°C and blocked in 3% BSA. Cells were incubated with primary antibodies specific for TAPT1 (Atlas Antibodies; HPA042567; 1/500), acetylated tubulin (Proteintech; 17711-1-AP; 1/1000) or γ tubulin (Proteintech; 66320-1-Ig; 1/1000) and corresponding secondary antibodies in blocking solution. Nuclei were counterstained with DAPI and coverslips were mounted in Mowiol. Images were acquired using an Olympus IX70 microscope with 60×1.42 NA oil-immersion lens, Exfo 120 metal halide illumination with excitation, dichroic and emission filters (Semrock,

Rochester, NY), and a Hamamatsu Orca-R2 camera (C10600-10B-H), controlled by Volocity 5.4.1 (Perkin Elmer). Chromatic shifts in images were registration corrected using TetraSpek fluorescent beads (Thermo Fisher Scientific). Images were acquired as 0.2 µm z-stacks and are presented as maximum projection images generated using image J. The brightness and contrast of all fluorescent images within the same experiment was adjusted in parallel for visualization but without obscuring or eliminating any information present in the original.

Steady-state and pulse-chase analyses of fibrillar collagen

For steady-state analysis, 2.5×10^4 cells/cm^2 were plated into 6-wells-plate and grown for 24 hours. Cells were then incubated for 2 hours with serum-free DMEM containing 4 mM glutamine, 100 µg/ml penicillin and streptomycin and 100 µg/ml (+)-sodium L-ascorbate (Sigma-Aldrich) to stimulate collagen production. Then, cells were labeled with ^{3}H-proline for 18 h. Type I collagen was extracted from medium and cell layer fractions and analyzed by SDS-urea-PAGE. For pulse-chase analysis, collagen labeling was performed for 4 h using 47.14 µCi of ^{3}H-proline/ml, then the labelling media was replaced with serum-free D-MEM containing 2 mM proline, 4 mM glutamine, 100 µg/ml penicillin and streptomycin and 100 µg/ml (+)-sodium L-ascorbate (chase media). Collagen was collected at 15, 30, 45 and 60 minutes after pulse. Samples were run on non-reducing SDS-urea-PAGE and the ratio between the densitometric value of collagen in the media and the total collagen (collagen present in medium and in cell layer) was evaluated to quantify the percentage of collagen secretion at each time point (Forlino *et al*, 2019). The experiments were performed in duplicates with technical triplicates each.

Determination of Protein Concentration

Collagen extraction and quantification from cell culture medium after 24 h and from decellularized matrix of fibroblasts after 7 days in culture was performed using Sircol^{TM} Soluble Collagen Assay (Biocolor) according to manufacturer's specifications. SFRP1 protein concentration in undiluted serum samples was measured using the ELISA Kit for Secreted Frizzled Related Protein 1 (SFRP1) (Cloud-Clone Corp.) according to manufacturer's specifications.

Electron microscopy

The structure of secreted collagen fibrils in human fibroblast supernatants was analysed by negative staining and transmission electron microscopy as described previously (Engel & Furthmayr, 1987). Five-microliter aliquots were adsorbed onto carbon-coated grids for 1 min, washed with two drops of water, and stained with two drops of 0.75% uranyl formate. The grids were rendered hydrophilic by glow discharge at low pressure in air. Specimens were observed in a Philips/FEI CM 100 BioTWIN transmission electron microscope operated at 60 kV accelerating voltage. Images were recorded with a side-mounted Olympus Veleta camera with a resolution of 2048 x 2048 pixels (2k x 2K).

Statistical analysis

Statistical analyses were performed using GraphPad Prism 5 software for experiments replicated at least three times. One way ANOVA followed by post hoc Bonferroni multiple comparison of patient to controls a, b or c was performed for the comparison across three control and one patient group. If only one group was available for the experimental set up, non-parametric Mann-Whitney-U test was performed. *P*-values < 0.05 (*), < 0.01 (**), < 0.001 (***) were considered to be statistically

significant. There was no randomization or blinding done for this study. One control sample was excluded from qPCR analysis due to insufficient RNA quality.

REFERENCES

Besio R, Garibaldi N, Leoni L, Cipolla L, Sabbioneda S, Biggiogera M, Mottes M, Aglan M, Otaify GA, Temtamy SA, *et al* (2019) Cellular stress due to impairment of collagen prolyl hydroxylation complex is rescued by the chaperone 4-phenylbutyrate. *Dis Model Mech* 12: dmm038521

Cintas HL, Siegel KL, Furst GP & Gerber LH (2003) Brief assessment of motor function: reliability and concurrent validity of the Gross Motor Scale. *Am J Phys Med Rehabil* 82: 33–41

Engel J & Furthmayr H (1987) Electron microscopy and other physical methods for the characterization of extracellular matrix components: Laminin, fibronectin, collagen IV, collagen VI, and proteoglycans. 145: 3–78

Forlino A, Tonelli F & Besio R (2019) Steady-state and pulse-chase analyses of fibrillar collagen. *Methods Mol Biol* 1952: 45–53

Jumper J, Evans R, Pritzel A, Green T, Figurnov M, Ronneberger O, Tunyasuvunakool K, Bates R, Žídek A, Potapenko A, *et al* (2021) Highly accurate protein structure prediction with AlphaFold. *Nat 2021 5967873* 596: 583–589

Koerber F, Schulze Uphoff U, Koerber S, Schönau E, Maintz D & Semler O (2012) Introduction of a new standardized assessment score of spine morphology in osteogenesis imperfecta. *Rofo* 184: 719–725

Pettersen EF, Goddard TD, Huang CC, Meng EC, Couch GS, Croll TI, Morris JH & Ferrin TE (2021) UCSF ChimeraX: Structure visualization for researchers,

educators, and developers. *Protein Sci* 30: 70

Pfaffl MW (2001) A new mathematical model for relative quantification in real-time RT-PCR. *Nucleic Acids Res* 29: 45e – 45

Pires DEV, Ascher DB & Blundell TL (2014) mCSM: predicting the effects of mutations in proteins using graph-based signatures. *Bioinformatics* 30: 335

Richards S, Aziz N, Bale S, Bick D, Das S, Gastier-Foster J, Grody WW, Hegde M, Lyon E, Spector E, *et al* (2015) Standards and guidelines for the interpretation of sequence variants: a joint consensus recommendation of the American College of Medical Genetics and Genomics and the Association for Molecular Pathology. *Genet Med* 17: 405–424

Ruck-Gibis J, Plotkin H, Hanley J & Wood-Dauphinee S (2001) Reliability of the Gross Motor Function Measure for children with osteogenesis imperfecta. *Pediatr Phys Ther* 13

Wang X, Spandidos A, Wang H & Seed B (2012) PrimerBank: a PCR primer database for quantitative gene expression analysis, 2012 update. *Nucleic Acids Res* 40: D1144
