# Supplementary figures and images for: TAPT1—at the crossroads of extracellular matrix and signaling in Osteogenesis imperfecta
Source: EMBO Mol Med. 2023 Jun 9;15(7):e17528. doi: 10.15252/emmm.202317528 (PMC10331569; doi:10.15252/emmm.202317528)

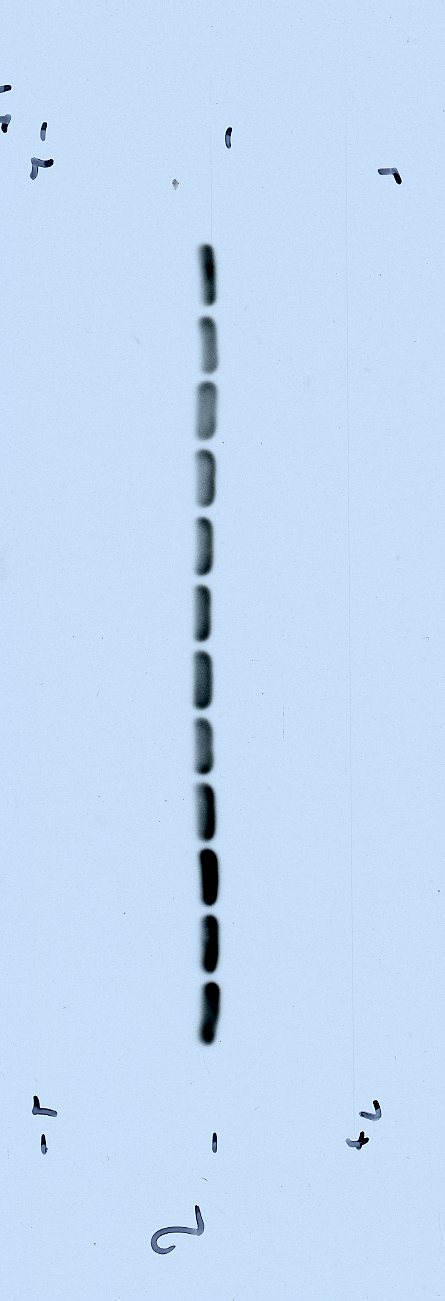

Supplement: Supplementary file 2 — Source Data for Figure 1 [file EMMM-15-e17528-s003.zip › Figure 1/1C/western_1C_GAPDH.jpg]

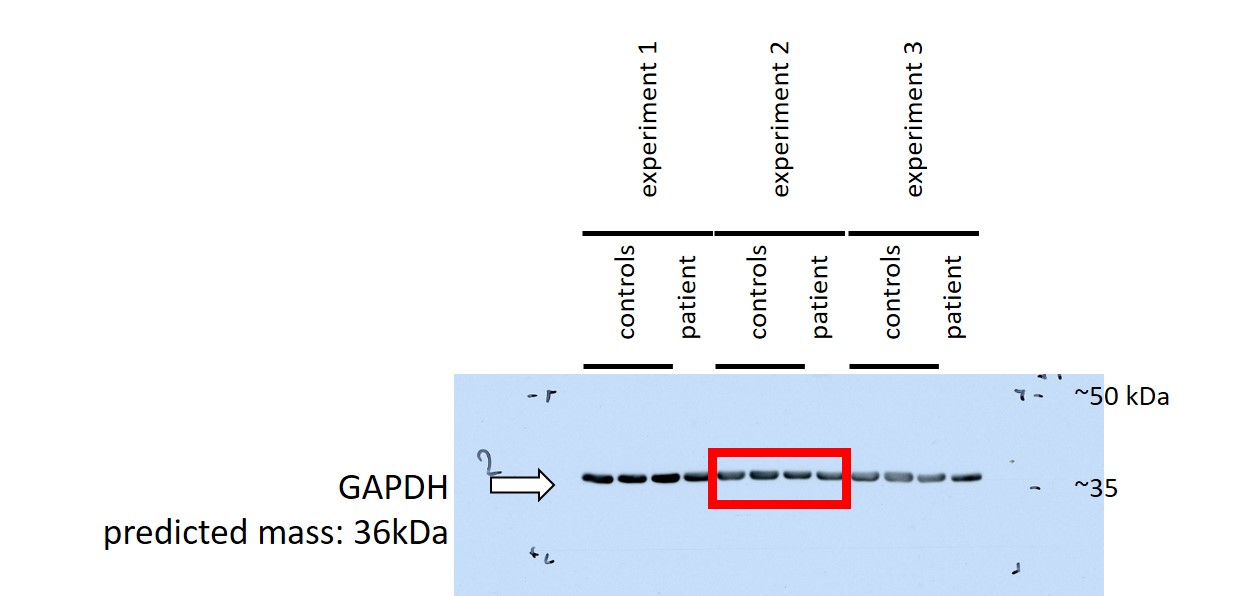

Supplement: Supplementary file 2 — Source Data for Figure 1 [file EMMM-15-e17528-s003.zip › Figure 1/1C/western_1C_GAPDH_annotated.jpg]

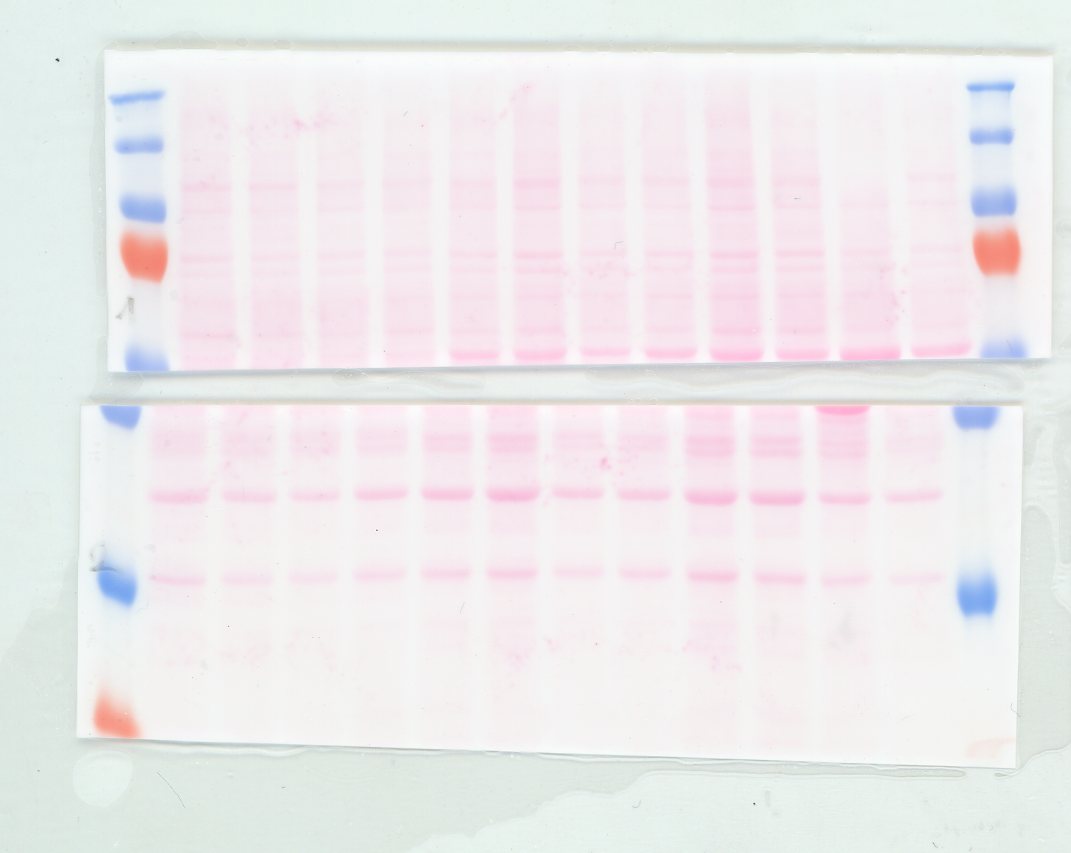

Supplement: Supplementary file 2 — Source Data for Figure 1 [file EMMM-15-e17528-s003.zip › Figure 1/1C/western_1C_Ponceau.jpg]

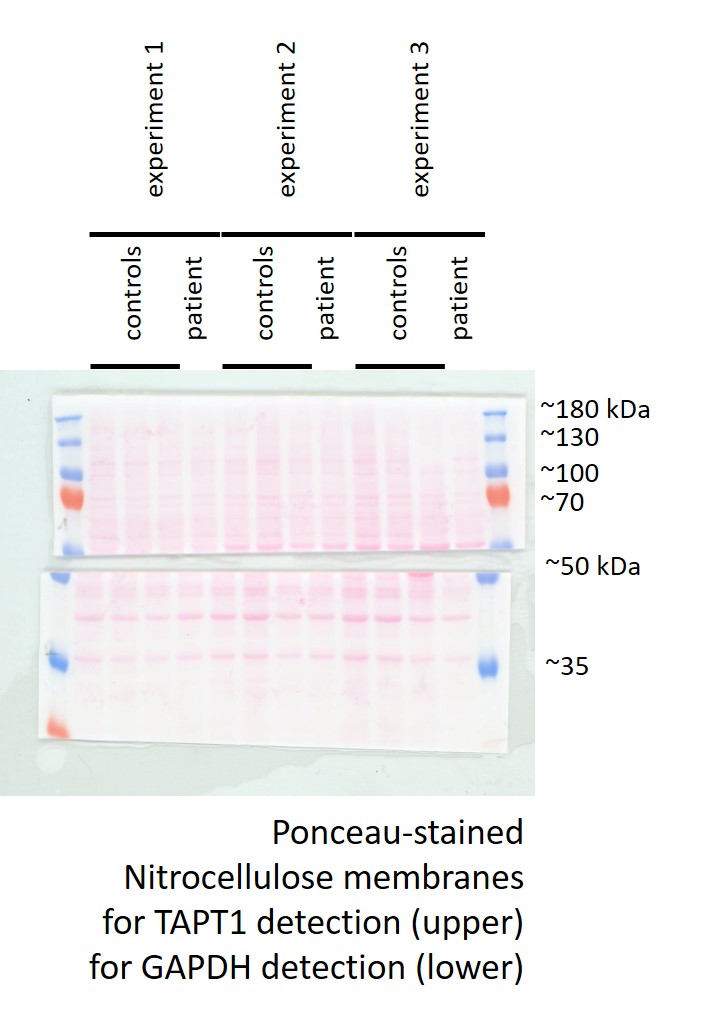

Supplement: Supplementary file 2 — Source Data for Figure 1 [file EMMM-15-e17528-s003.zip › Figure 1/1C/western_1C_Ponceau_annotated.jpg]

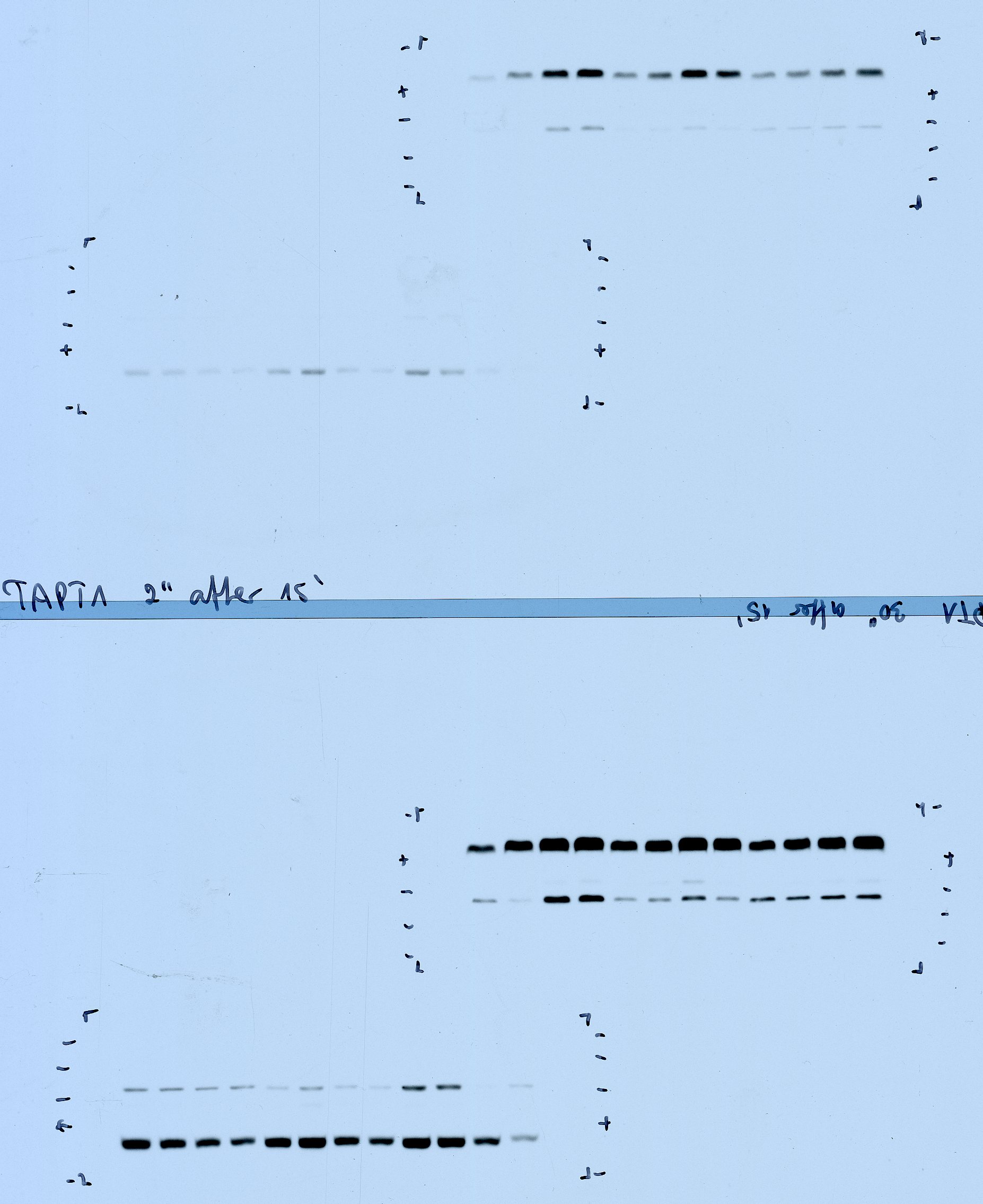

Supplement: Supplementary file 2 — Source Data for Figure 1 [file EMMM-15-e17528-s003.zip › Figure 1/1C/western_1C_TAPT1.jpg]

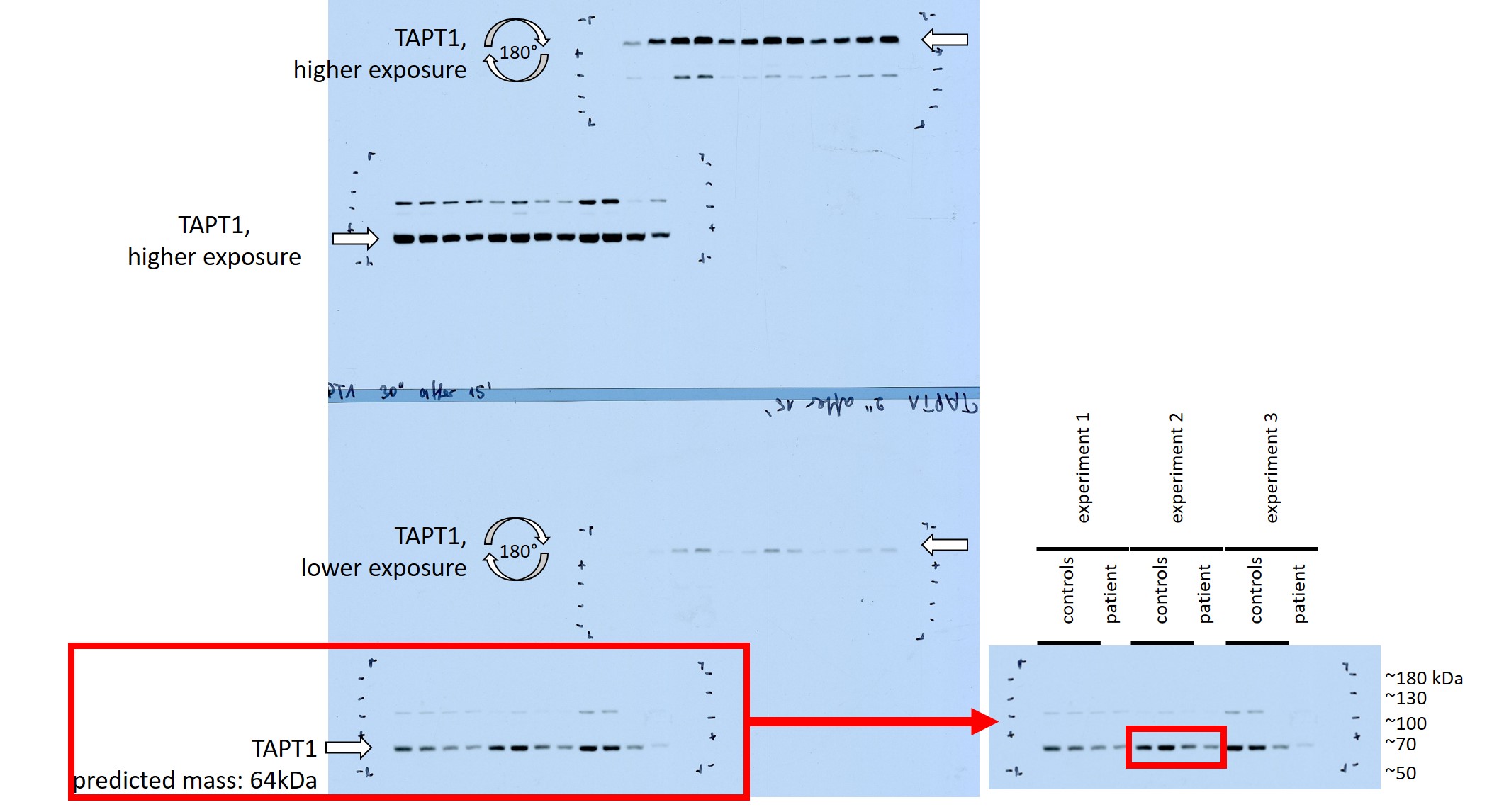

Supplement: Supplementary file 2 — Source Data for Figure 1 [file EMMM-15-e17528-s003.zip › Figure 1/1C/western_1C_TAPT1_annotated.jpg]

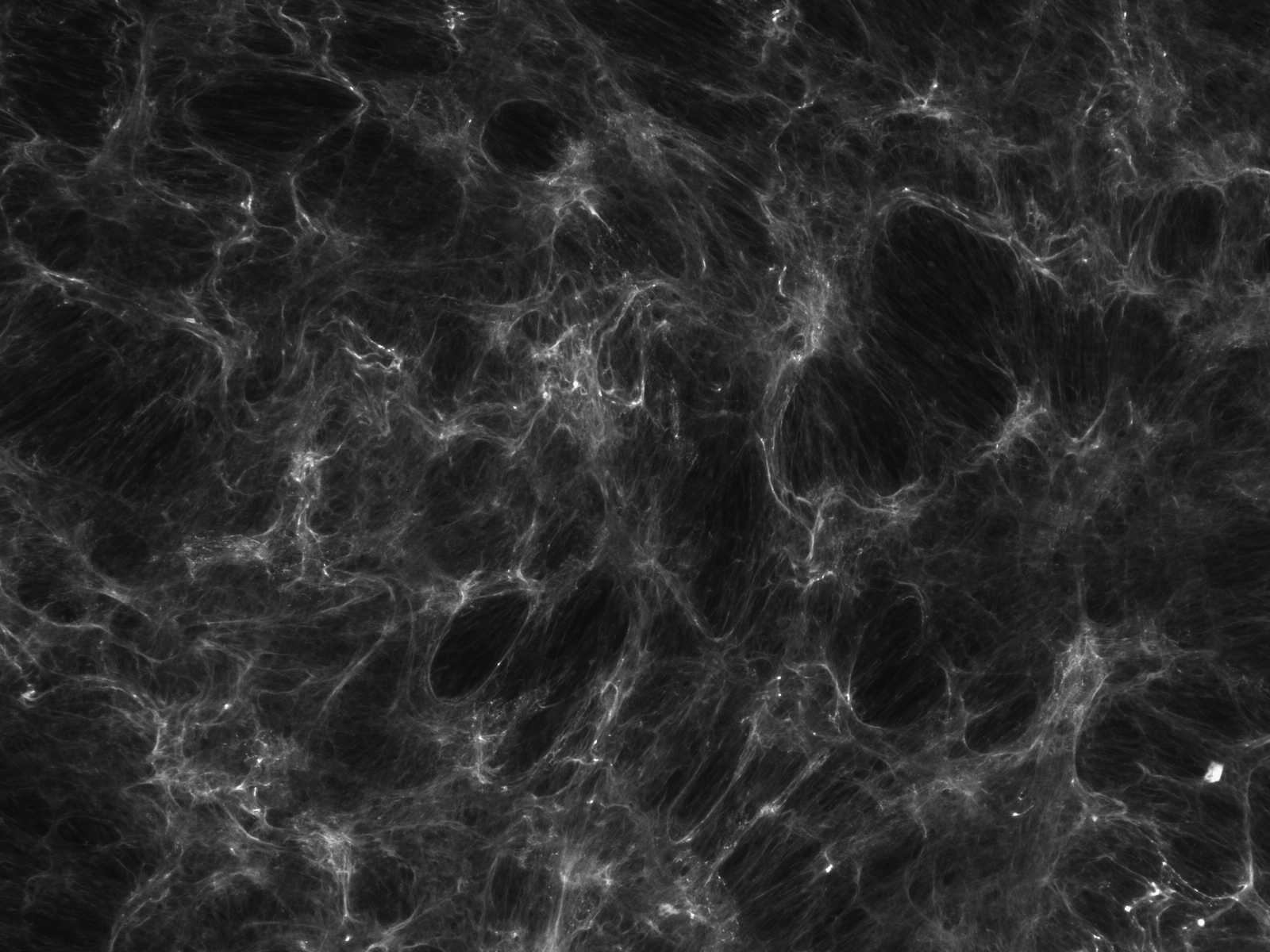

Supplement: Supplementary file 2 — Source Data for Figure 1 [file EMMM-15-e17528-s003.zip › Figure 1/1E/IF_1E_control_COL I.tif]

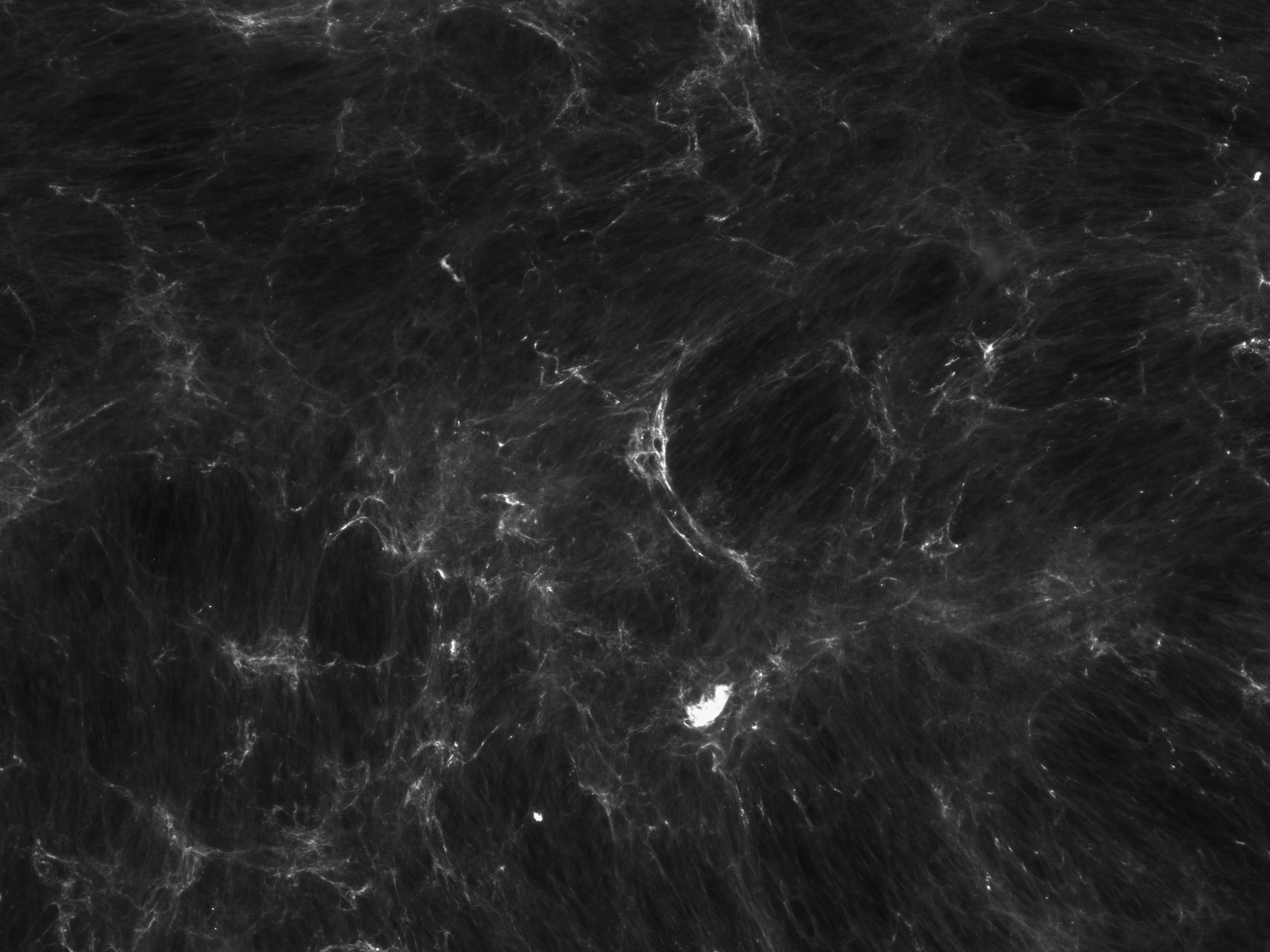

Supplement: Supplementary file 2 — Source Data for Figure 1 [file EMMM-15-e17528-s003.zip › Figure 1/1E/IF_1E_patient_COL I.tif]

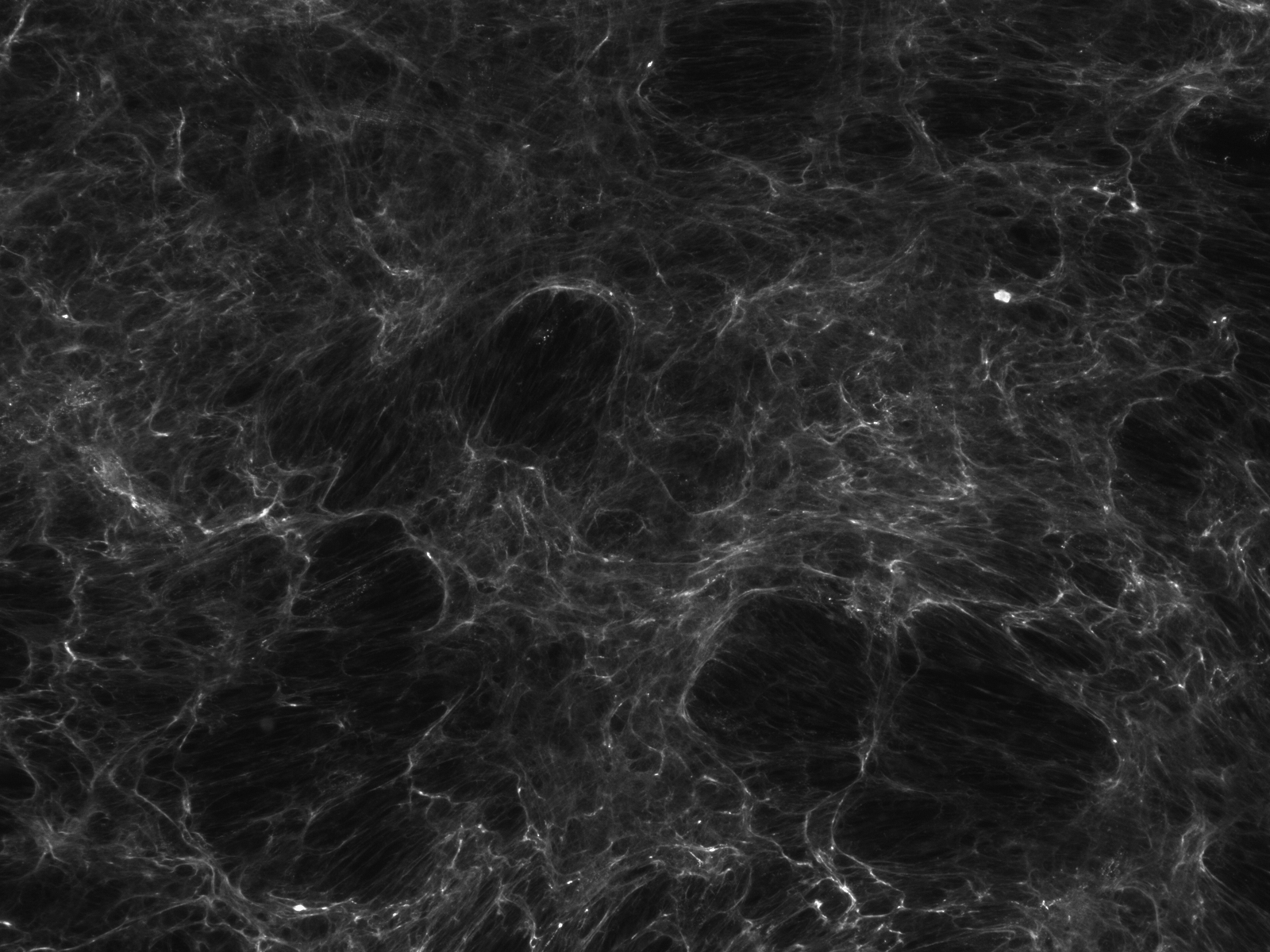

Supplement: Supplementary file 2 — Source Data for Figure 1 [file EMMM-15-e17528-s003.zip › Figure 1/1E/IF_1E_replicate/IF_1E_control2_COL I.tif]

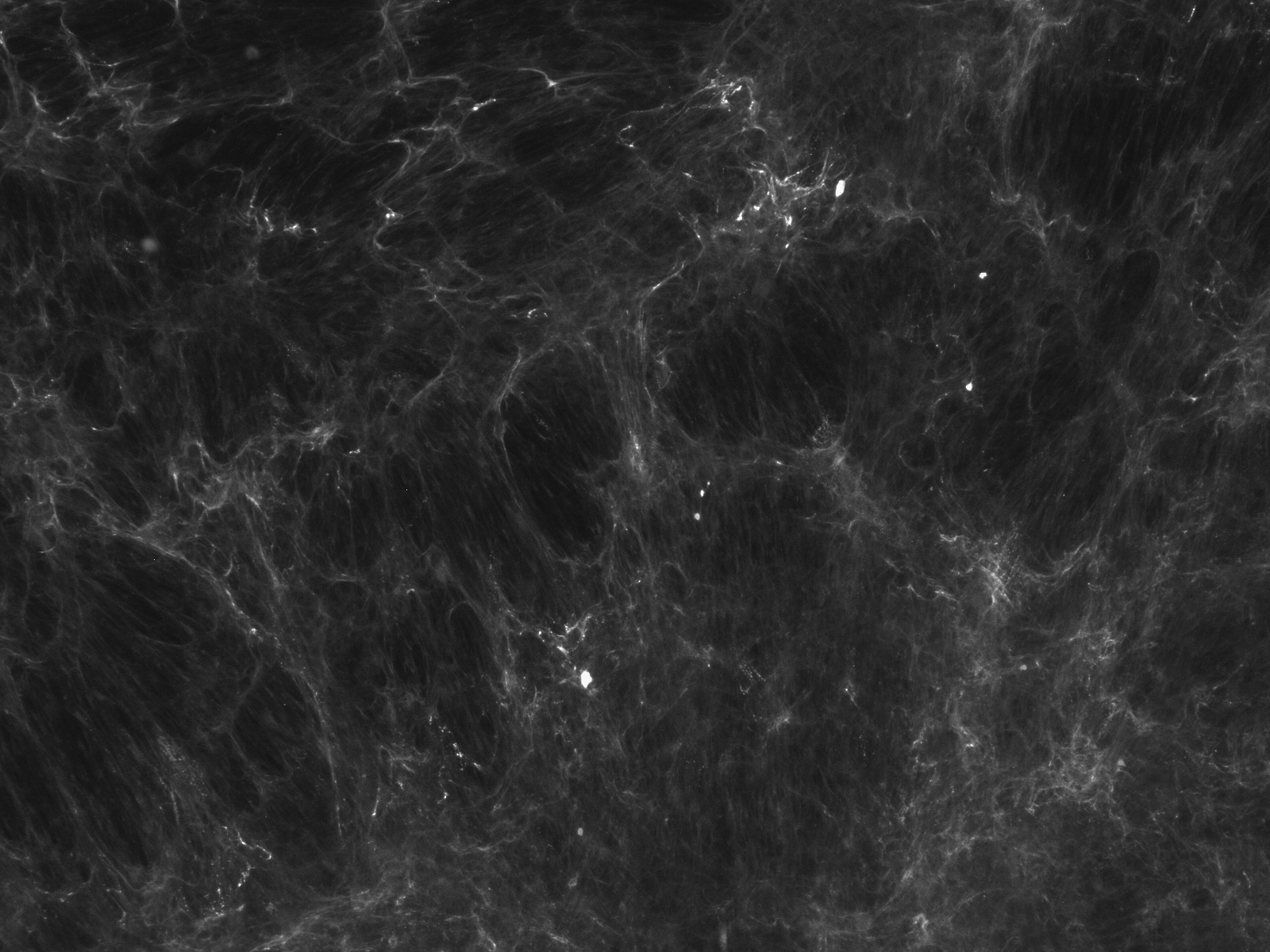

Supplement: Supplementary file 2 — Source Data for Figure 1 [file EMMM-15-e17528-s003.zip › Figure 1/1E/IF_1E_replicate/IF_1E_control2_COL I_replicate.tif]

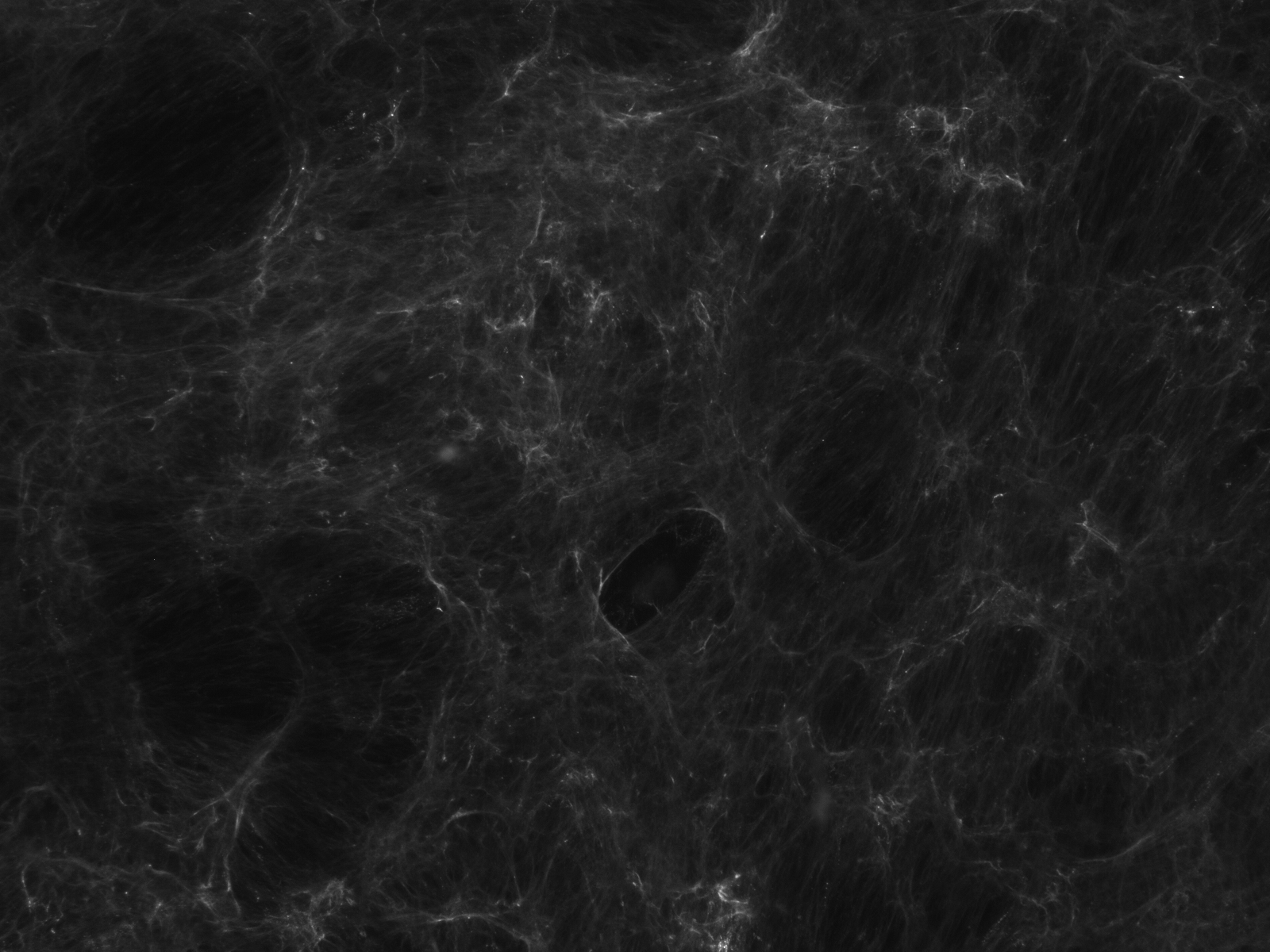

Supplement: Supplementary file 2 — Source Data for Figure 1 [file EMMM-15-e17528-s003.zip › Figure 1/1E/IF_1E_replicate/IF_1E_control2_COL I_replicate2.tif]

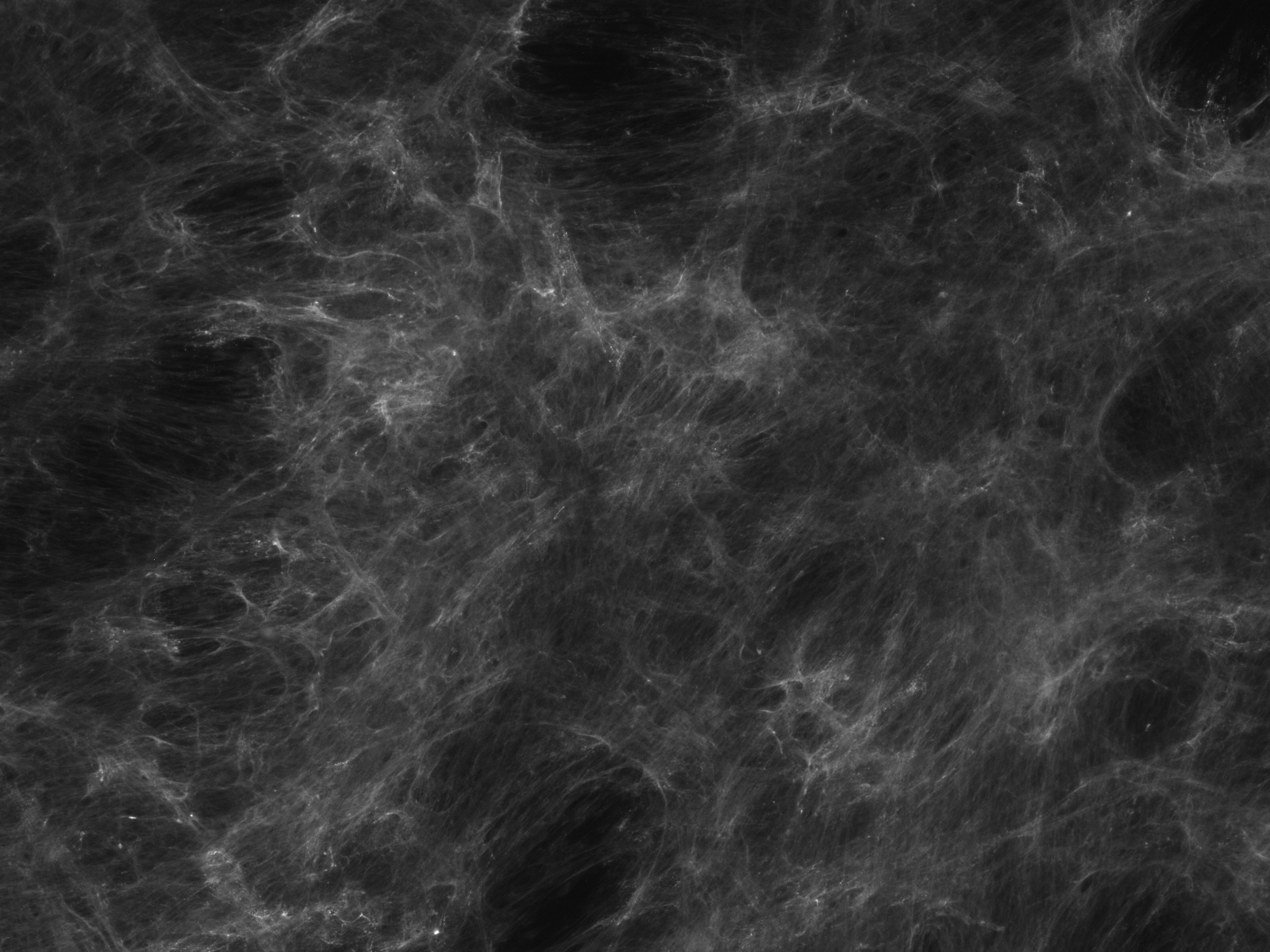

Supplement: Supplementary file 2 — Source Data for Figure 1 [file EMMM-15-e17528-s003.zip › Figure 1/1E/IF_1E_replicate/IF_1E_control3_COL I.tif]

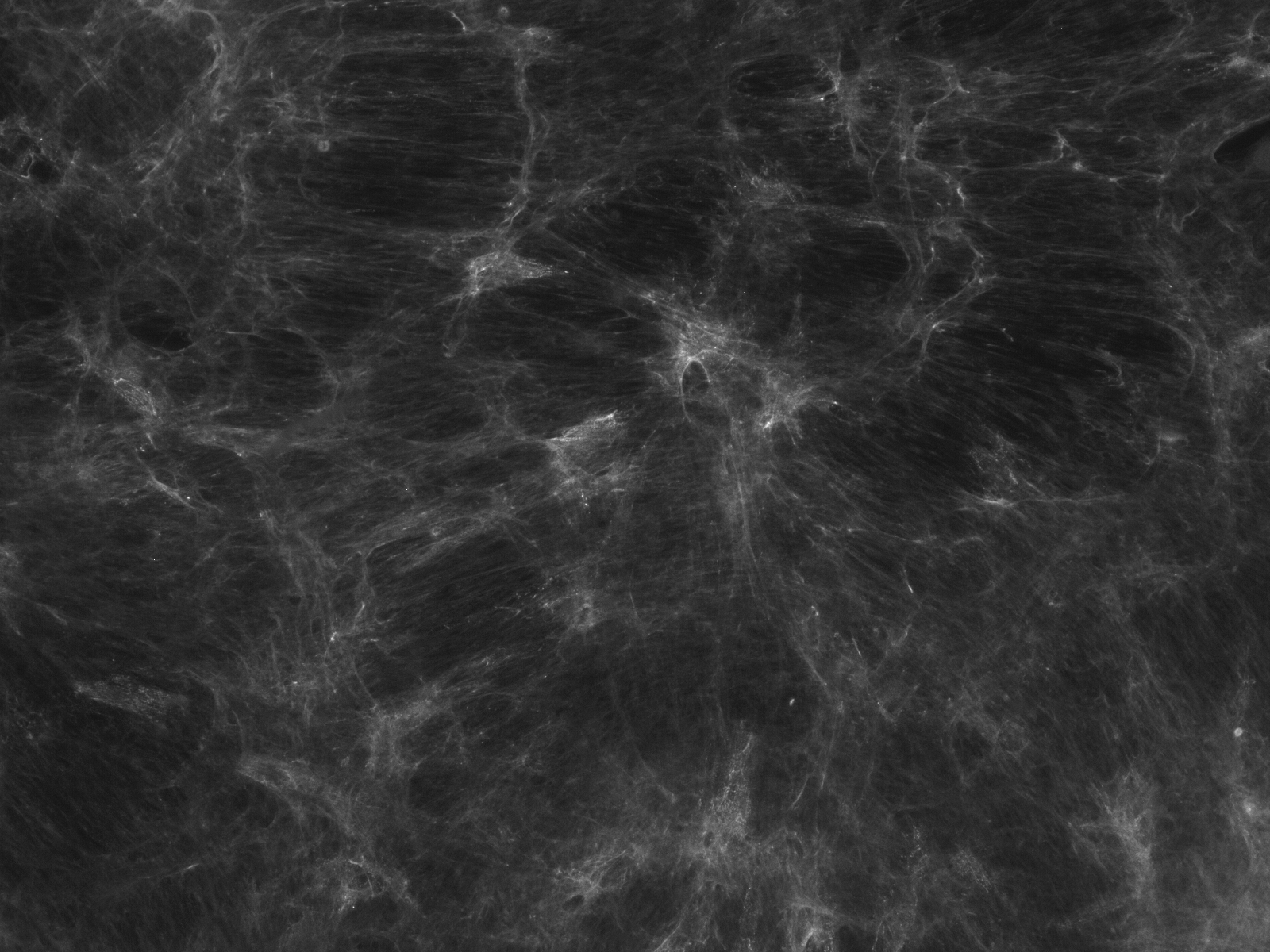

Supplement: Supplementary file 2 — Source Data for Figure 1 [file EMMM-15-e17528-s003.zip › Figure 1/1E/IF_1E_replicate/IF_1E_control3_COL I_replicate.tif]

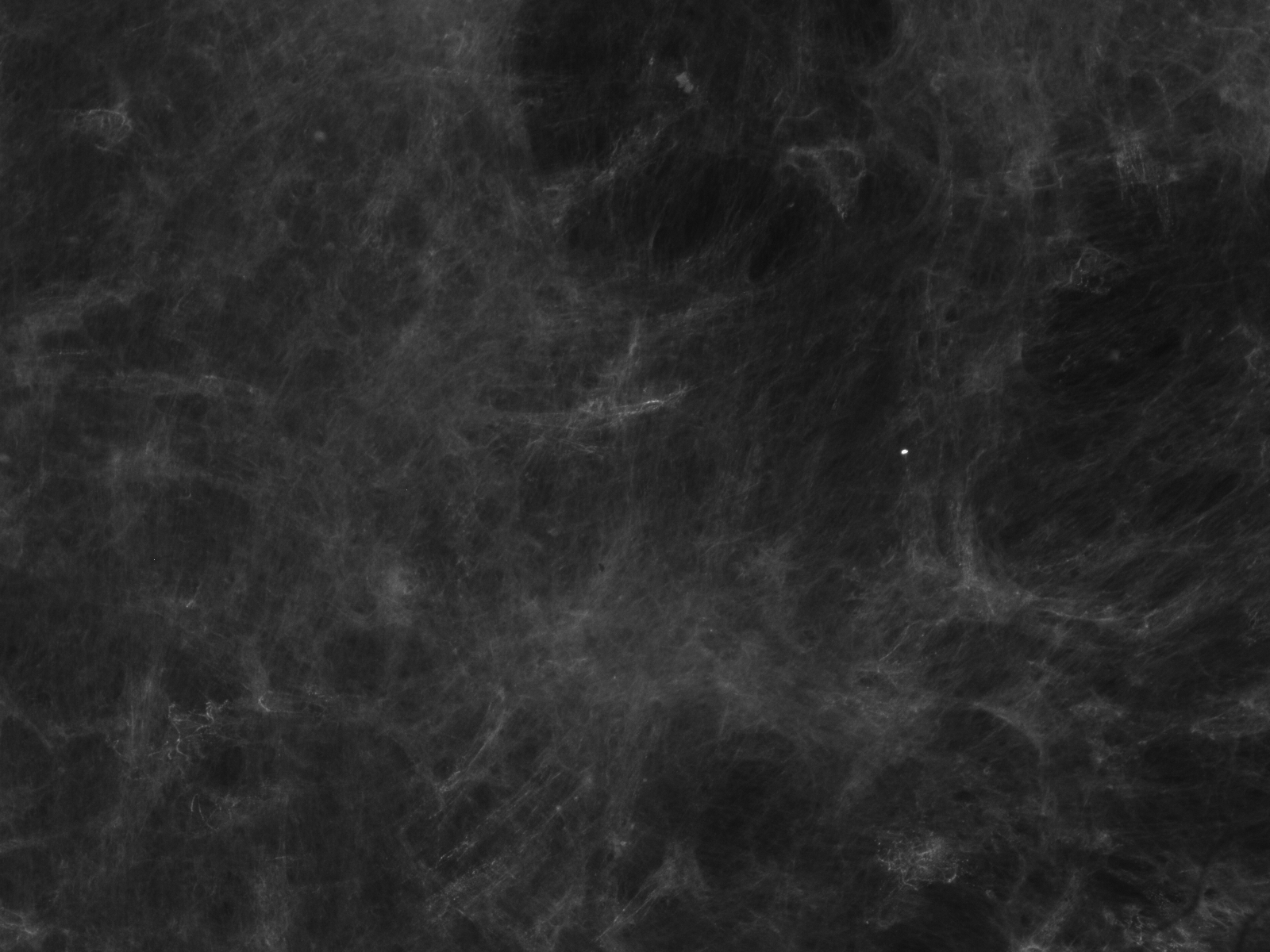

Supplement: Supplementary file 2 — Source Data for Figure 1 [file EMMM-15-e17528-s003.zip › Figure 1/1E/IF_1E_replicate/IF_1E_control3_COL I_replicate2.tif]

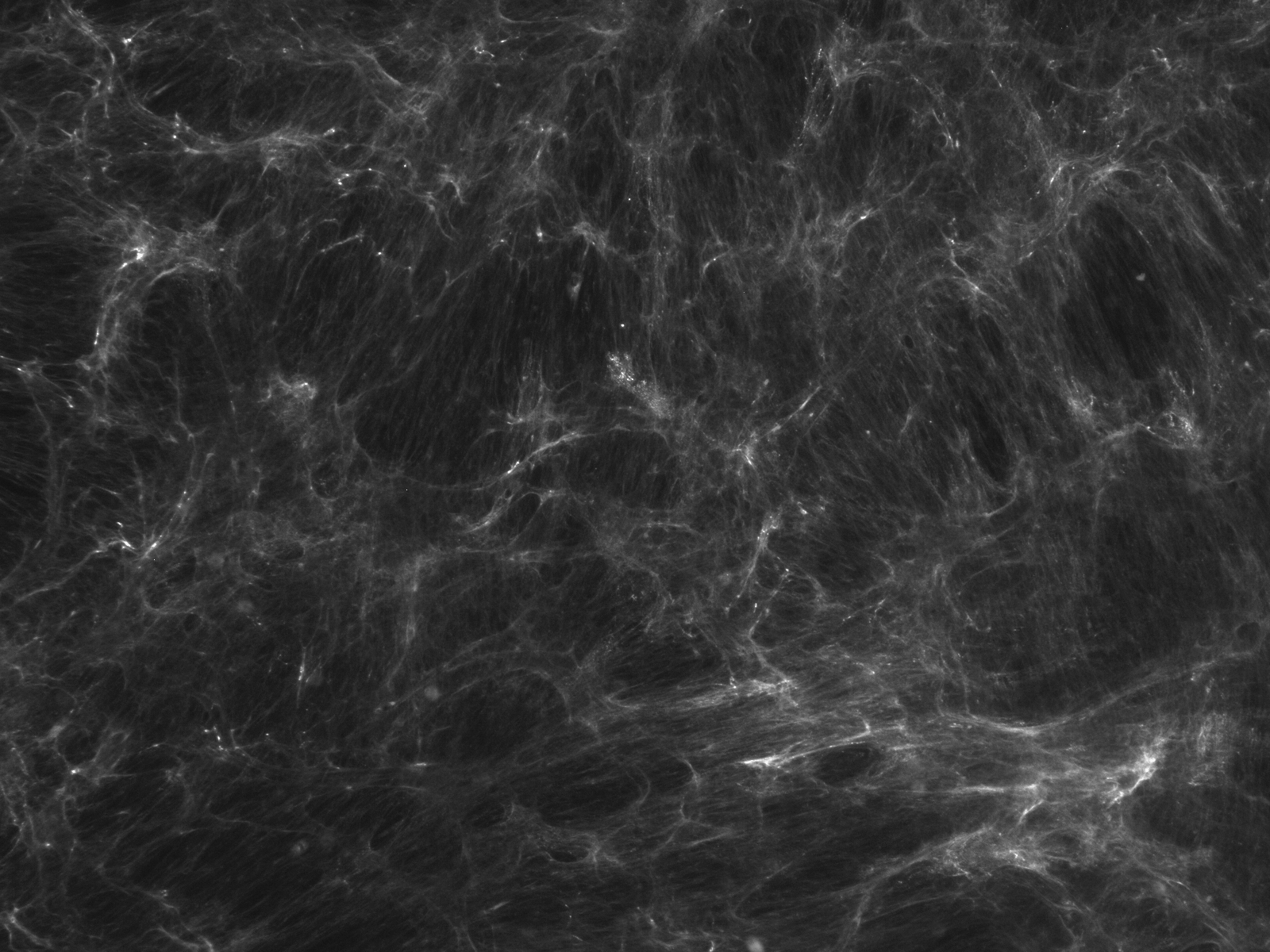

Supplement: Supplementary file 2 — Source Data for Figure 1 [file EMMM-15-e17528-s003.zip › Figure 1/1E/IF_1E_replicate/IF_1E_control_COL I_replicate.tif]

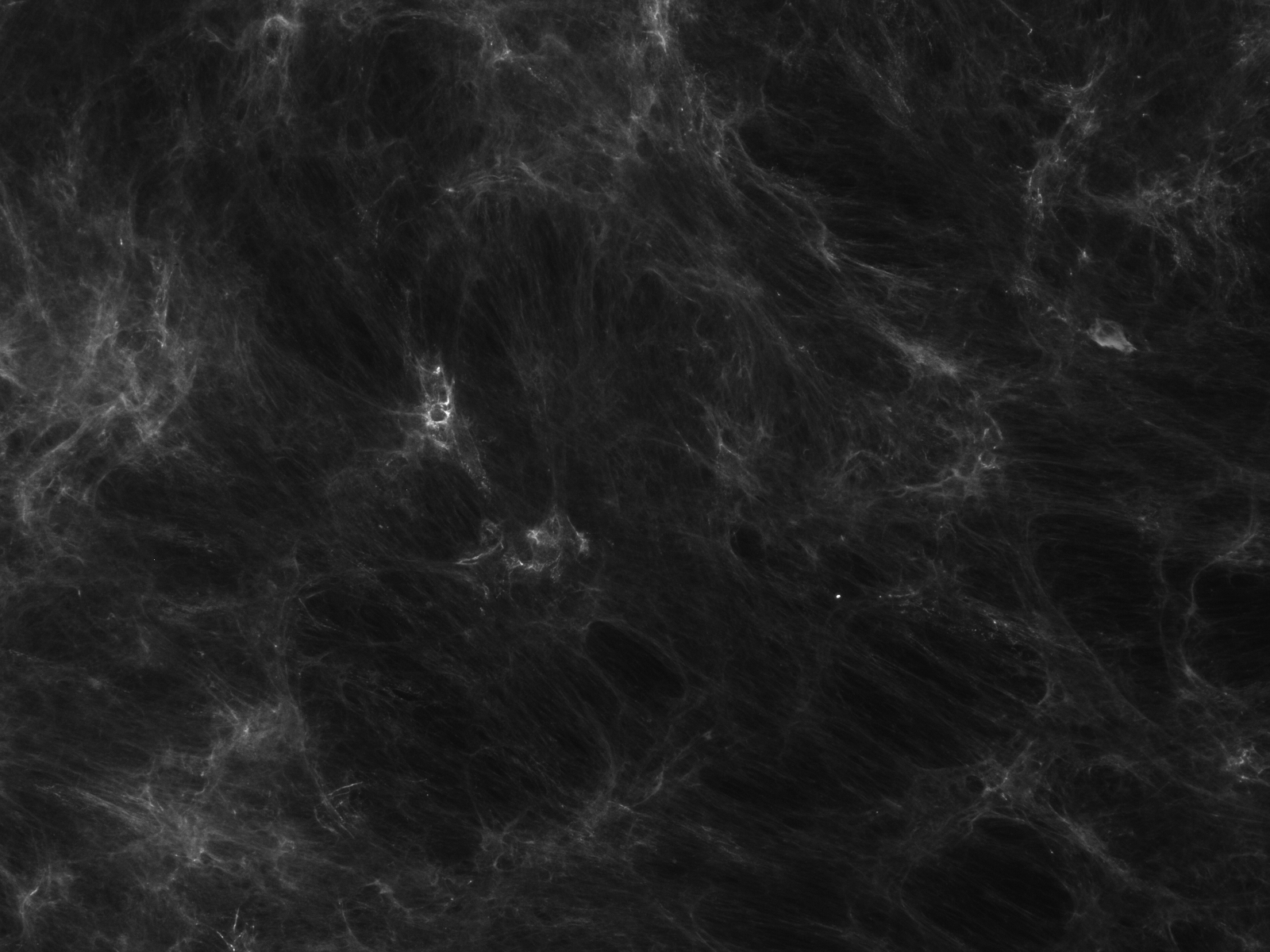

Supplement: Supplementary file 2 — Source Data for Figure 1 [file EMMM-15-e17528-s003.zip › Figure 1/1E/IF_1E_replicate/IF_1E_control_COL I_replicate2.tif]

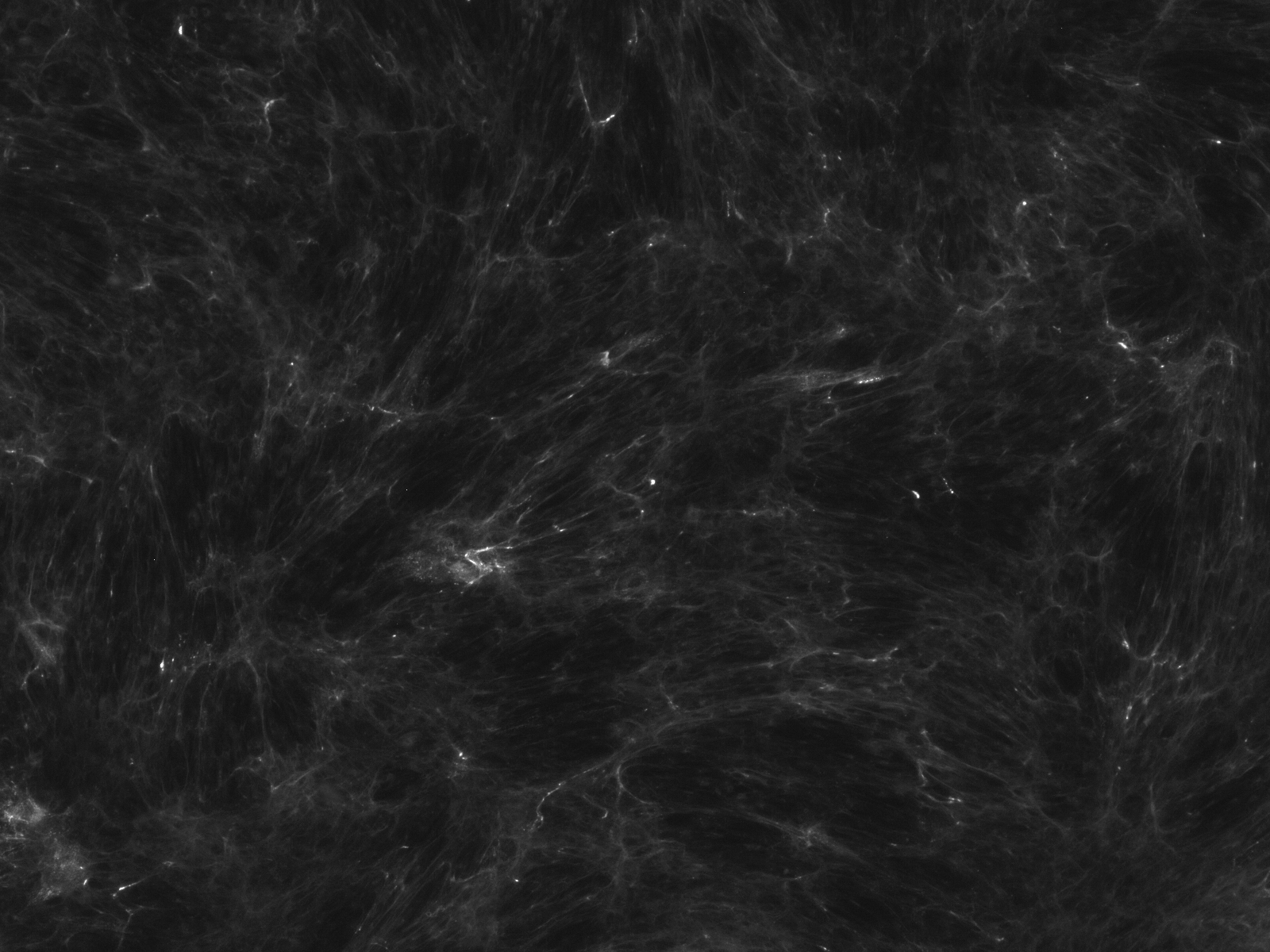

Supplement: Supplementary file 2 — Source Data for Figure 1 [file EMMM-15-e17528-s003.zip › Figure 1/1E/IF_1E_replicate/IF_1E_patient_COL I_replicate.tif]

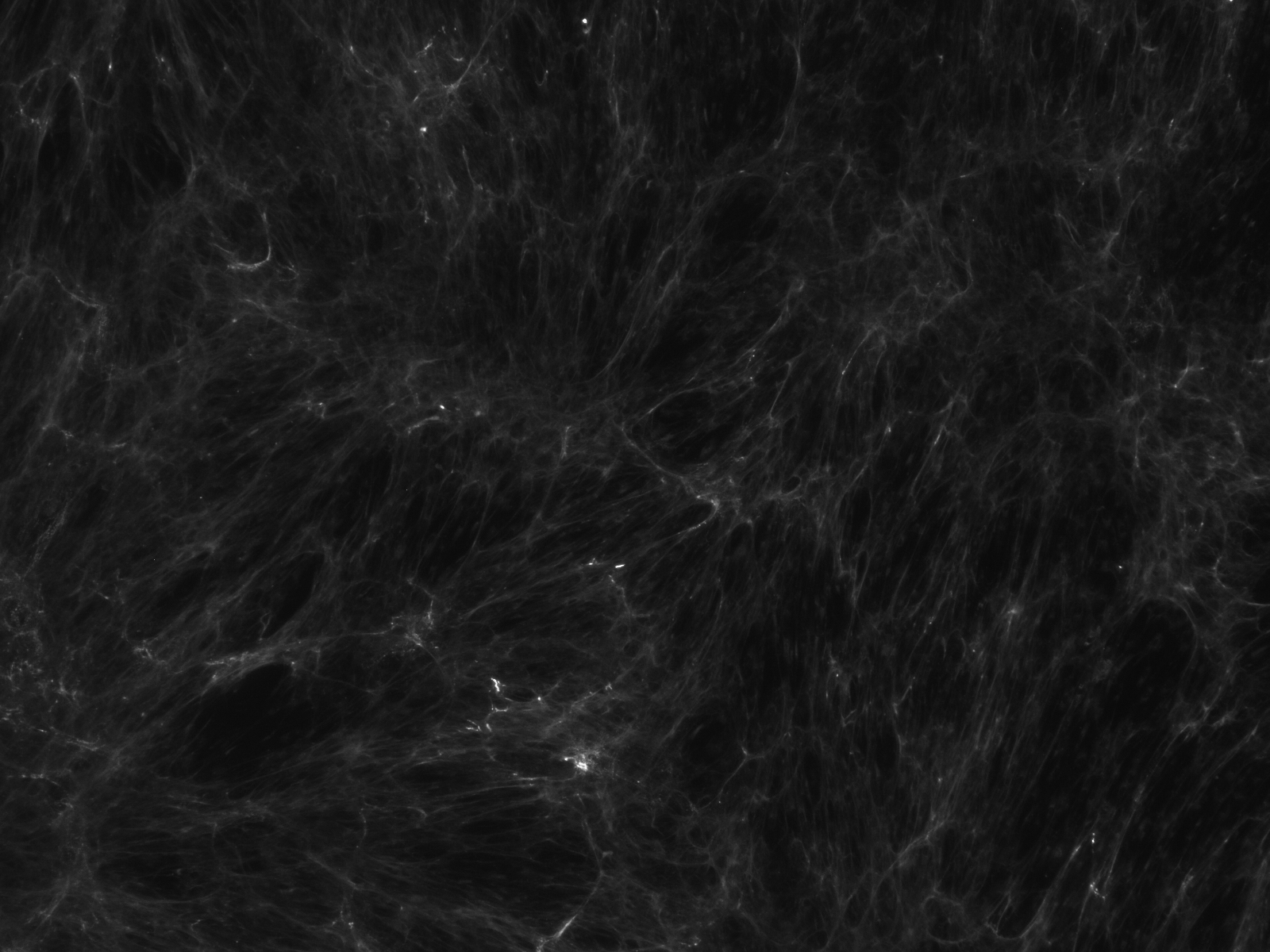

Supplement: Supplementary file 2 — Source Data for Figure 1 [file EMMM-15-e17528-s003.zip › Figure 1/1E/IF_1E_replicate/IF_1E_patient_COL I_replicate2.tif]

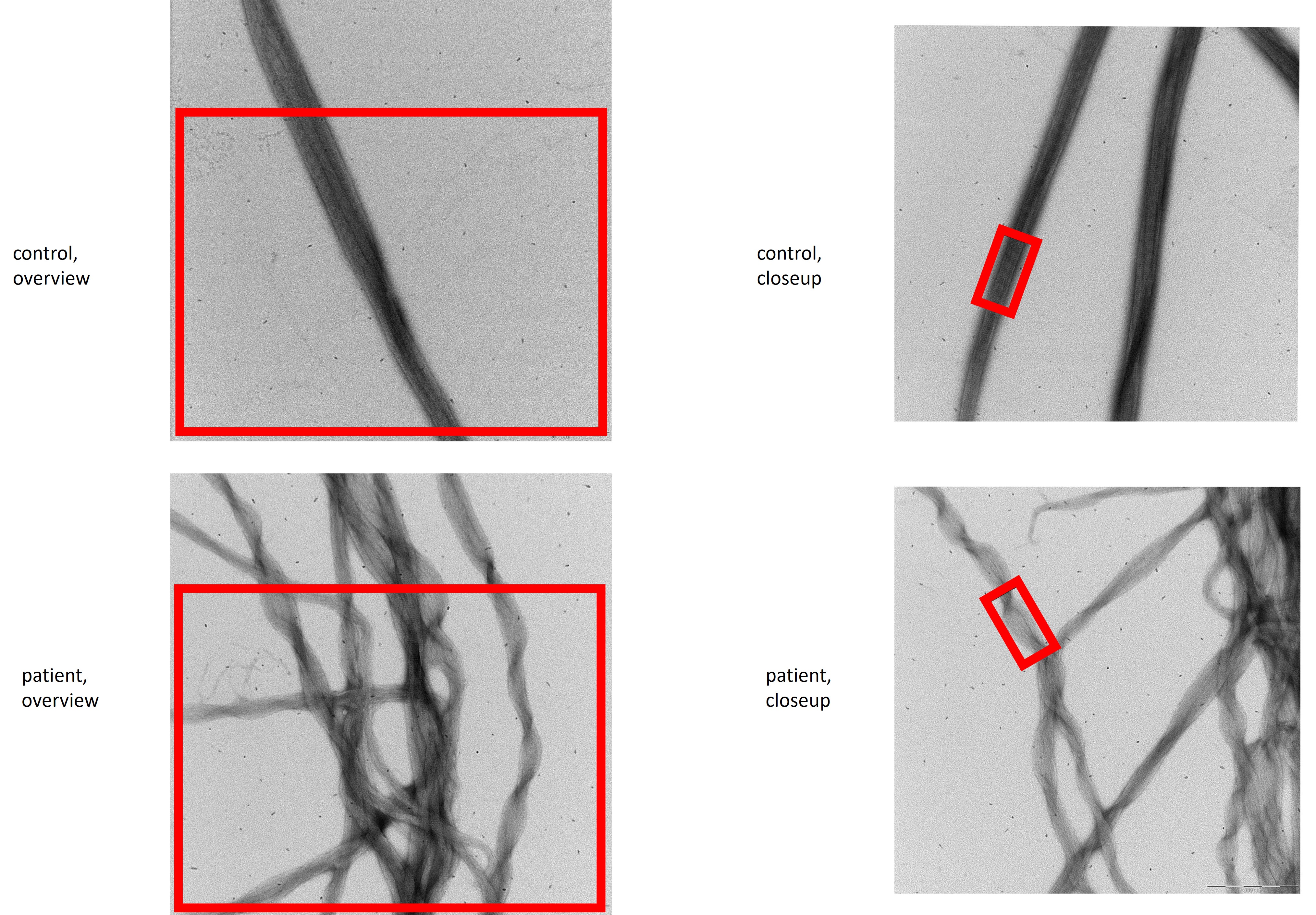

Supplement: Supplementary file 2 — Source Data for Figure 1 [file EMMM-15-e17528-s003.zip › Figure 1/1F/EM_1F_annotated.jpg]

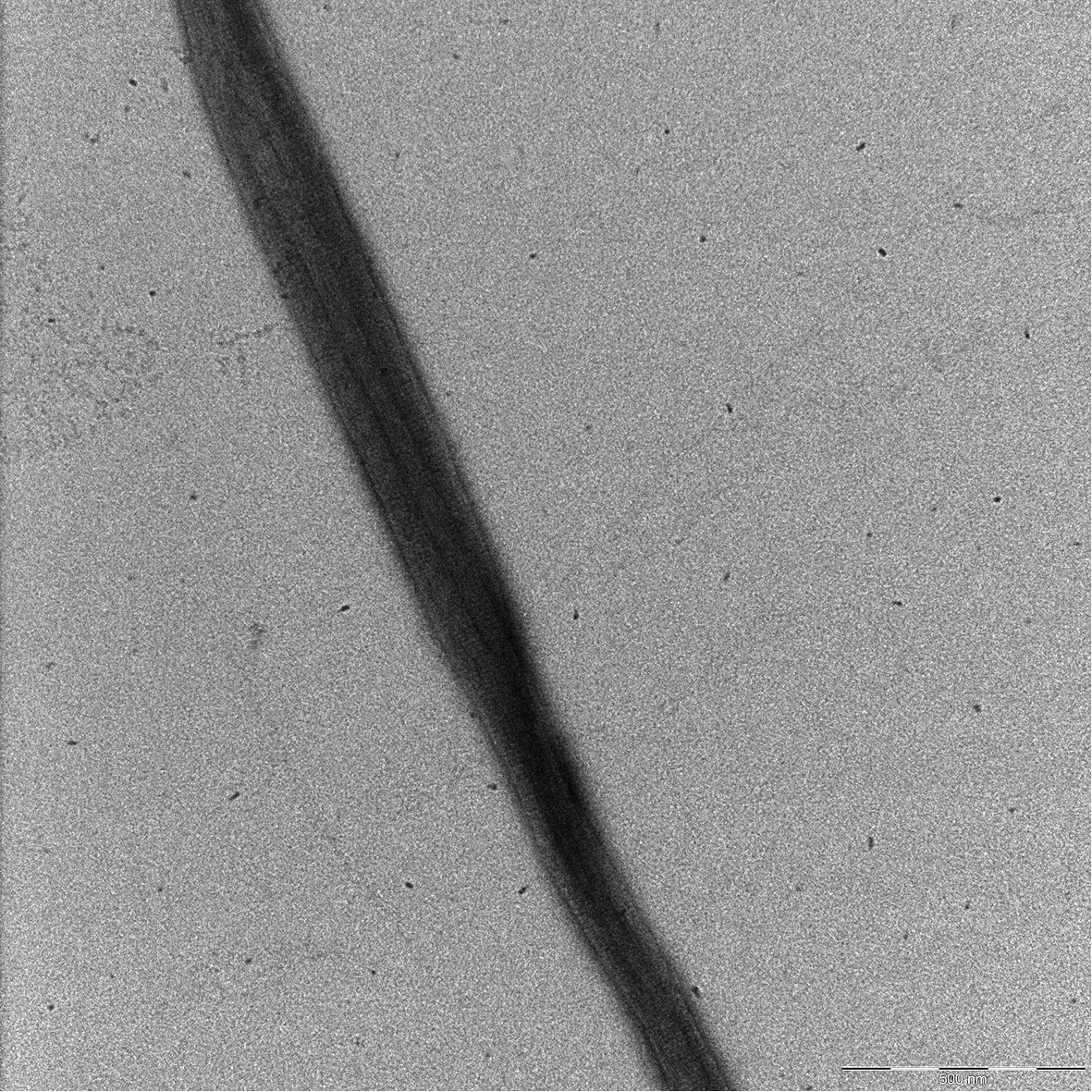

Supplement: Supplementary file 2 — Source Data for Figure 1 [file EMMM-15-e17528-s003.zip › Figure 1/1F/EM_1F_control.jpg]

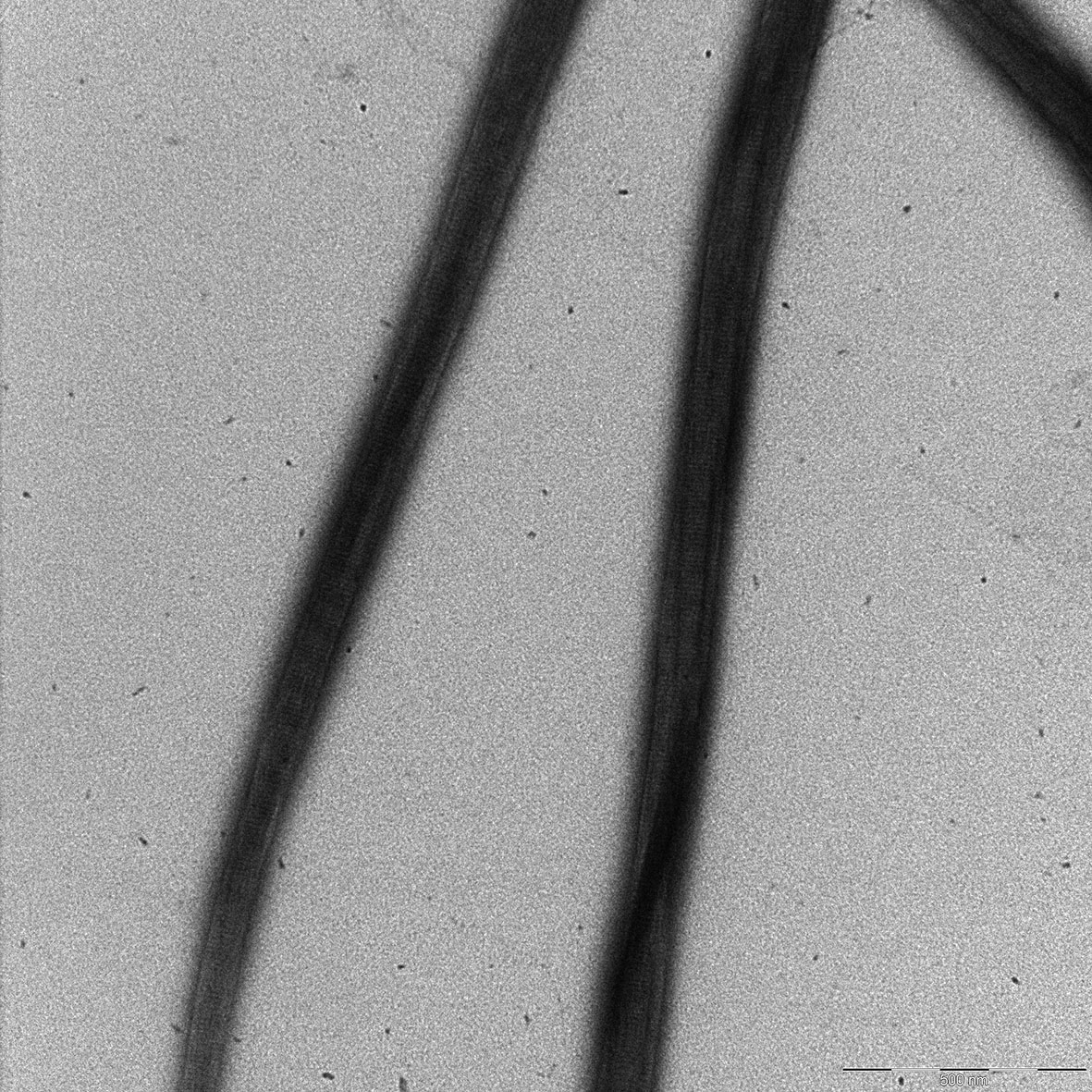

Supplement: Supplementary file 2 — Source Data for Figure 1 [file EMMM-15-e17528-s003.zip › Figure 1/1F/EM_1F_control_insert.jpg]

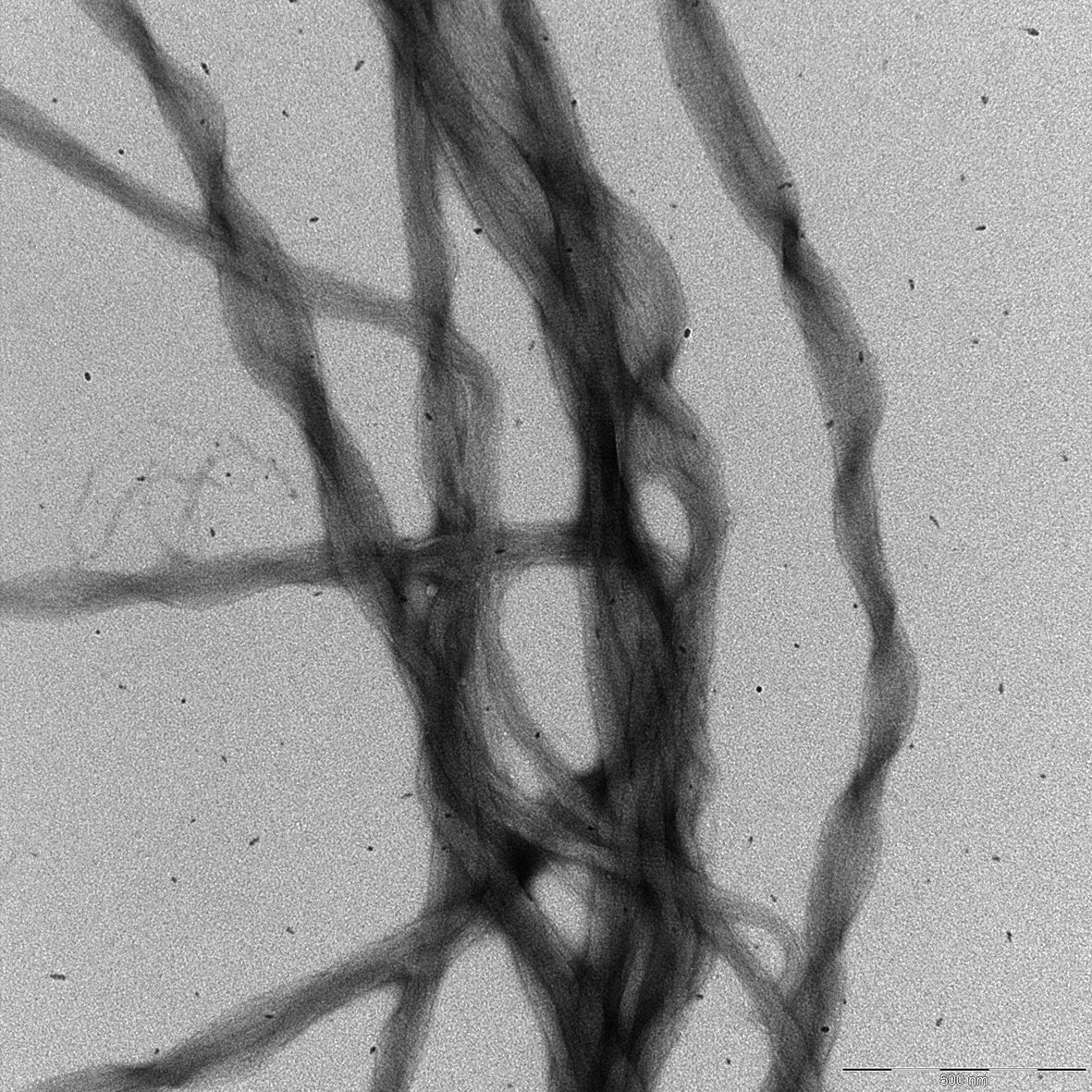

Supplement: Supplementary file 2 — Source Data for Figure 1 [file EMMM-15-e17528-s003.zip › Figure 1/1F/EM_1F_patient.jpg]

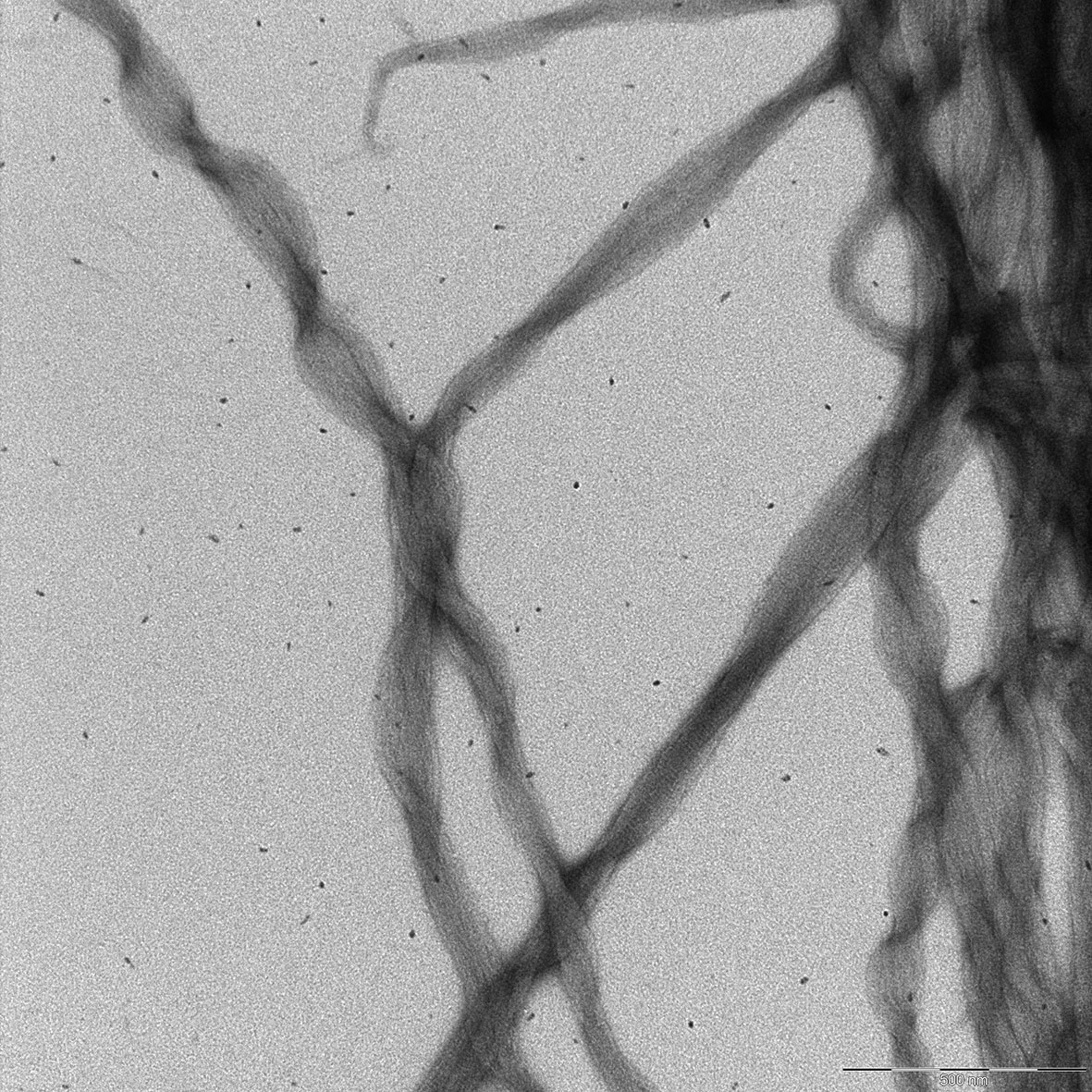

Supplement: Supplementary file 2 — Source Data for Figure 1 [file EMMM-15-e17528-s003.zip › Figure 1/1F/EM_1F_patient_insert.jpg]

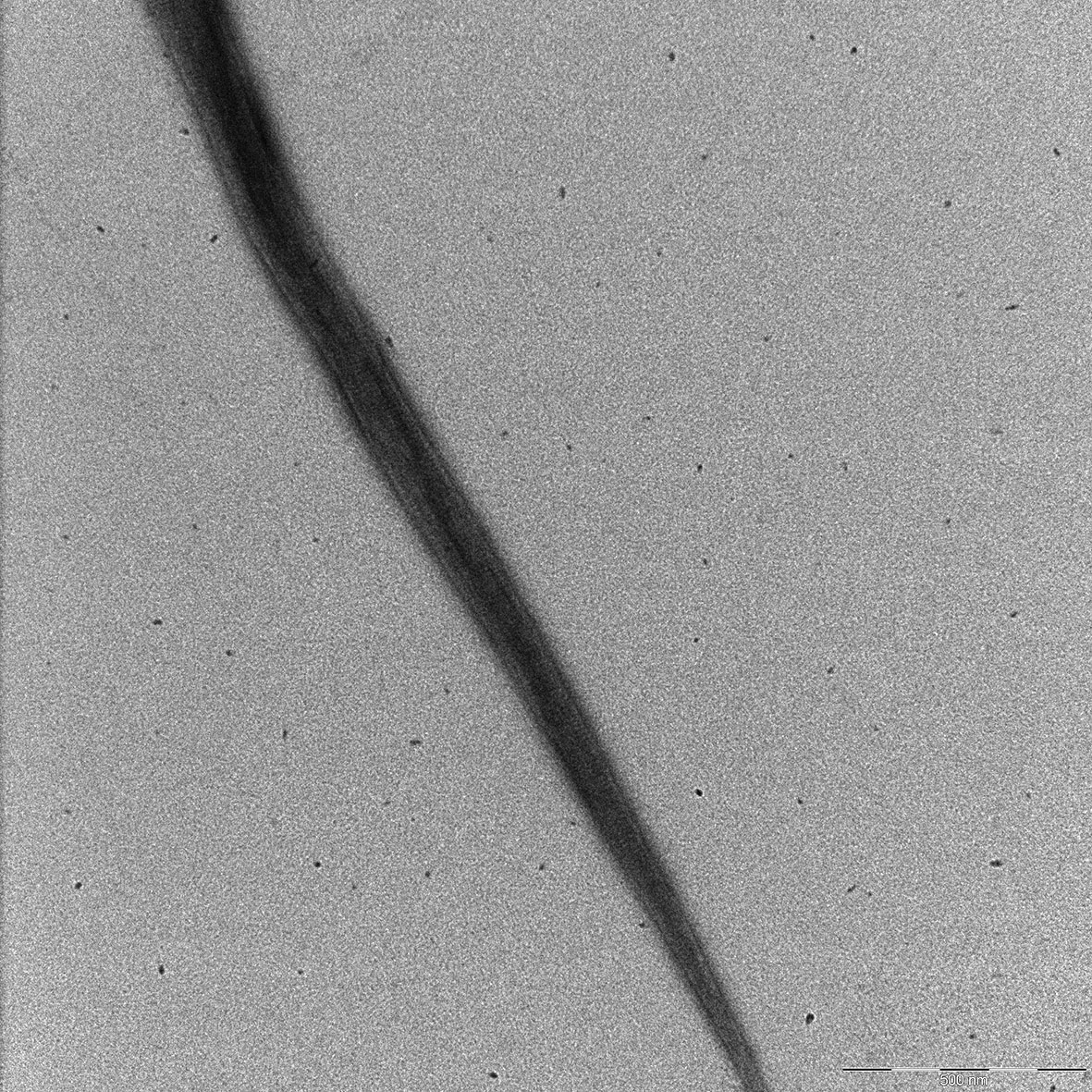

Supplement: Supplementary file 2 — Source Data for Figure 1 [file EMMM-15-e17528-s003.zip › Figure 1/1F/EM_1F_replicate/EM_1F_control_replicate.jpg]

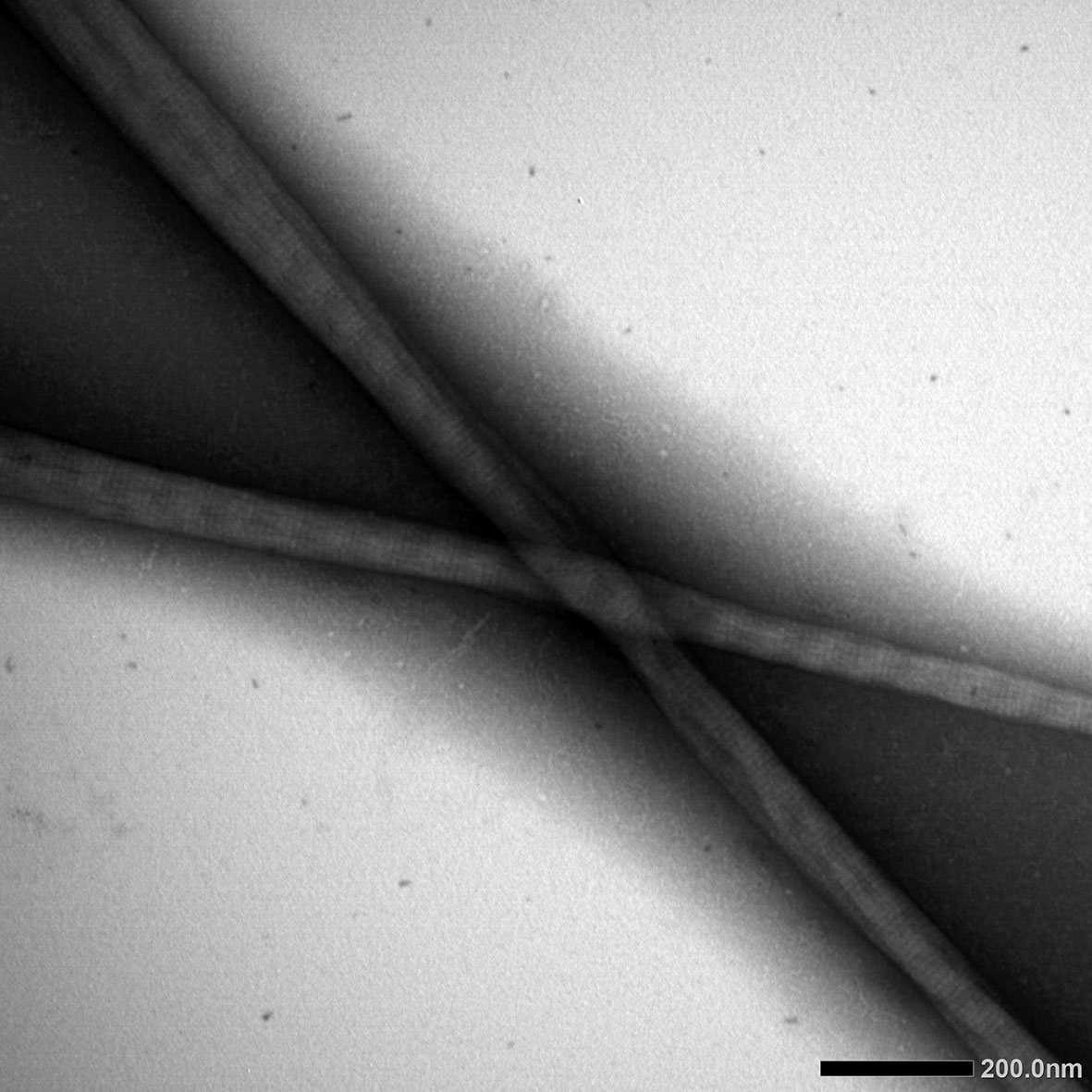

Supplement: Supplementary file 2 — Source Data for Figure 1 [file EMMM-15-e17528-s003.zip › Figure 1/1F/EM_1F_replicate/EM_1F_control_replicate_2.tif]

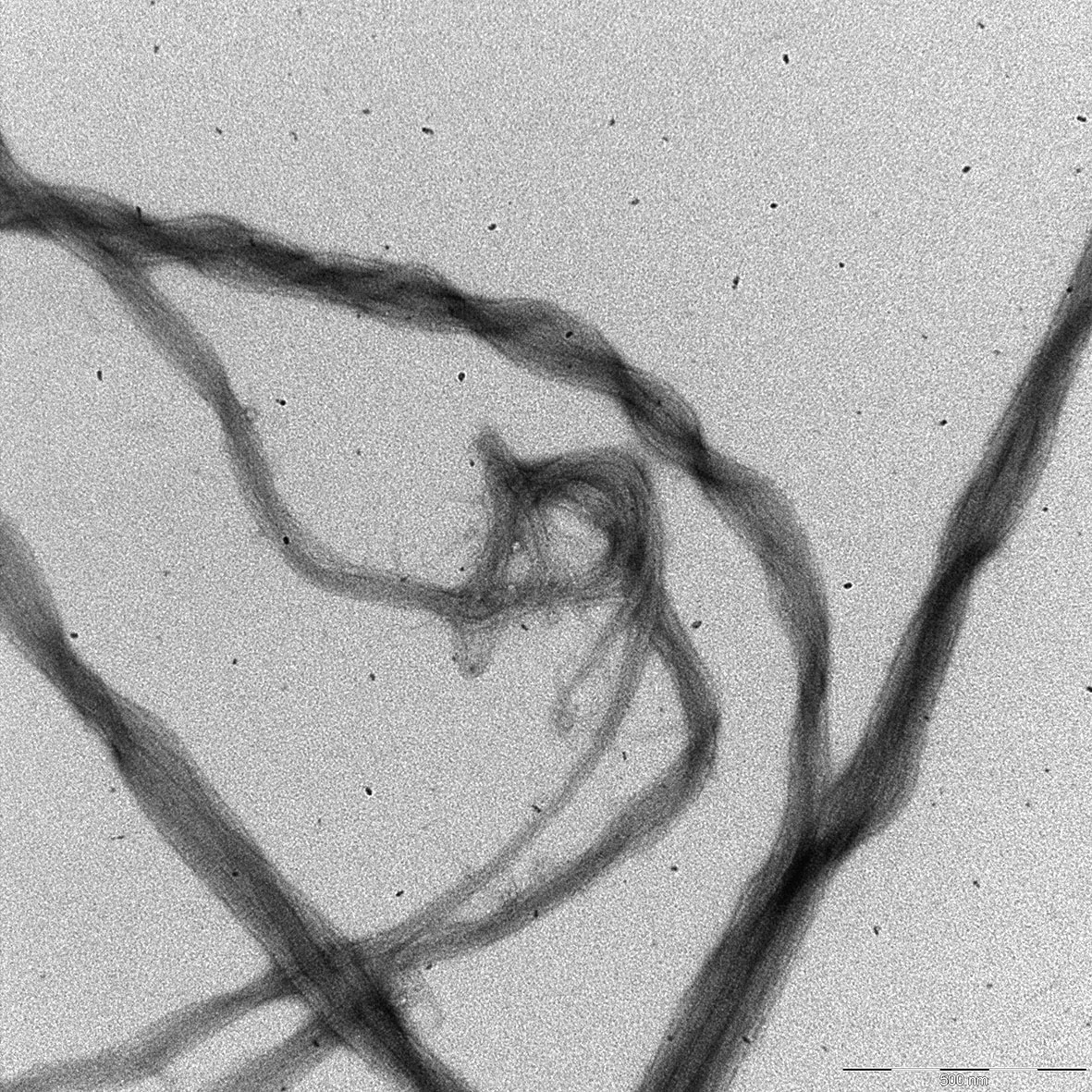

Supplement: Supplementary file 2 — Source Data for Figure 1 [file EMMM-15-e17528-s003.zip › Figure 1/1F/EM_1F_replicate/EM_1F_patient_replicate.jpg]

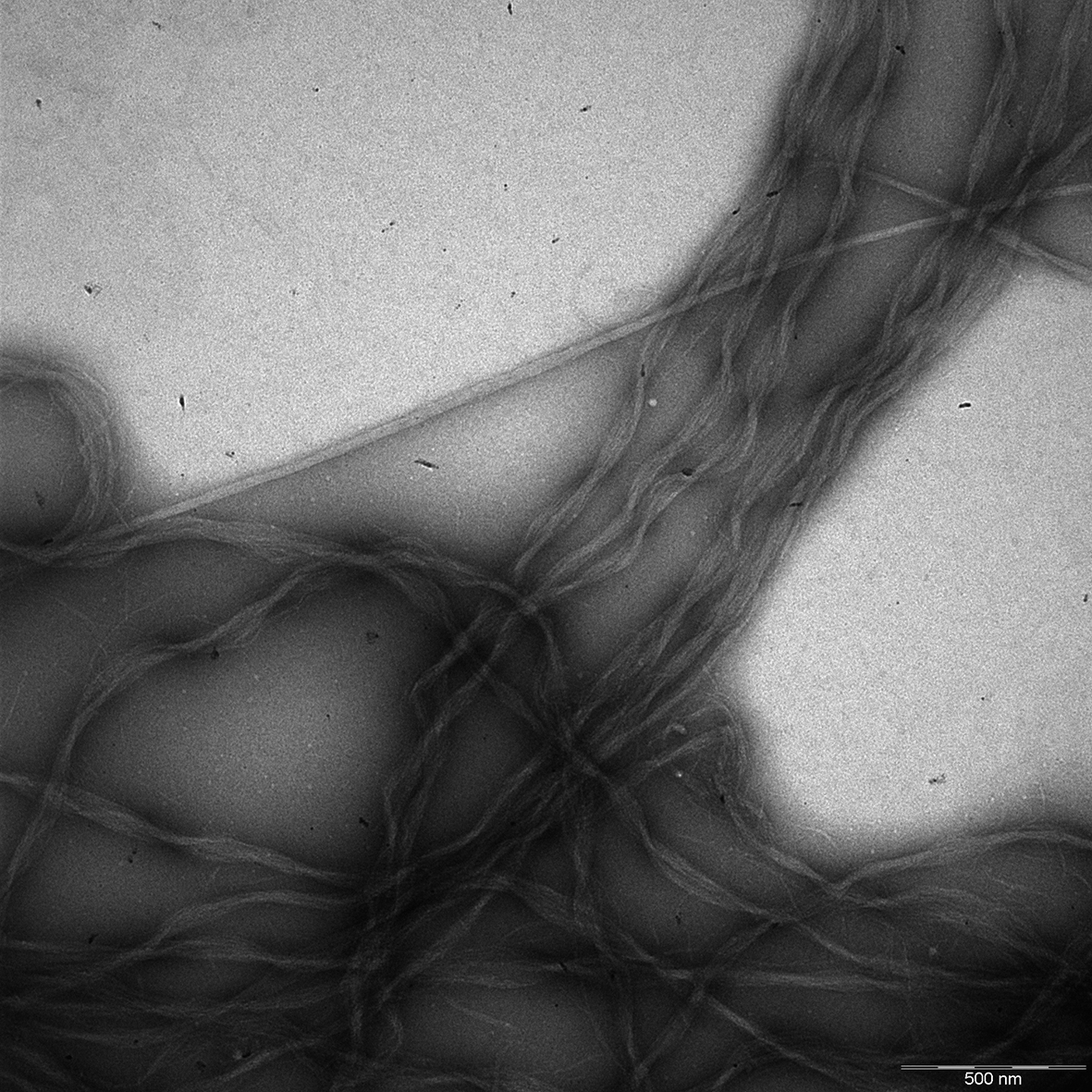

Supplement: Supplementary file 2 — Source Data for Figure 1 [file EMMM-15-e17528-s003.zip › Figure 1/1F/EM_1F_replicate/EM_1F_patient_replicate_2.tif]
