# Supplementary material for: TAPT1—at the crossroads of extracellular matrix and signaling in Osteogenesis imperfecta
Source: EMBO Mol Med. 2023 Jun 9;15(7):e17528. doi: 10.15252/emmm.202317528 (PMC10331569; doi:10.15252/emmm.202317528)
Supplement: Supplementary file 3 [file EMMM-15-e17528-s002.pdf]

EMBO Molecular Medicine

TAPT1 - at the crossroads of extracellular matrix and signalling in Osteogenesis imperfecta

Julia Etich, Jörg Oliver Semler, Nicola Stevenson, Alice Stephan, Roberta Besio, Nadia Garibaldi, Nadine Reintjes, Claudia Dafinger, Max Liebau, Ulrich Baumann, Matthias Mörgelin, Antonella Forlino, David Stephens, Christian Netzer, Frank Zaucke, and Mirko Rehberg
DOI: 10.15252/emmm.202317528

Corresponding authors: Mirko Rehberg (mirko.rehberg@uk-koeln.de) , Julia Etich (Julia.etich@uni-koeln.de)

Review Timeline:

Submission Date:	3rd Mar 23
Editorial Decision:	24th Mar 23
Revision Received:	16th May 23
Accepted:	25th May 23

Editor: Zeljko Durdevic

Transaction Report:

(Note: With the exception of the correction of typographical or spelling errors that could be a source of ambiguity, letters and reports are not edited. Depending on transfer agreements, referee reports obtained elsewhere may or may not be included in this compilation. Referee reports are anonymous unless the Referee chooses to sign their reports.)

24th Mar 2023

Dear Dr. Rehberg,

Thank you for the submission of your manuscript to EMBO Molecular Medicine. We have now received feedback from one reviewer who we asked to evaluate your manuscript. As you will see from the report, the referee recognizes potential interest of the study but also raises important concerns that should be addressed in a revision of the current manuscript. Point #2 should be addressed experimentally and all the other points textually. For Correspondence articles, Expanded View material can in principle only be in the form of structured data or dataset files, however, as the additional figures and detailed material and methods are in our view important for the study, please make the Expanded View file to "Appendix" with a table of content and rename figures to "Appendix Figure S1" etc. Please callout the figures in the main text. Please check our Author Guidelines for more information about manuscript preparation. https://www.embopress.org/page/journal/17574684/authorguide

Addressing the reviewers' concerns in full will be necessary for further considering the manuscript in our journal. EMBO Molecular Medicine encourages a single round of revision only and therefore, acceptance or rejection of the manuscript will depend on the completeness of your responses included in the next, final version of the manuscript. For this reason, and to save you from any frustrations in the end, I would strongly advise against returning an incomplete revision.

Please also amend the following:

Submit a complete Author Checklist. https://www.embopress.org/pb-assets/embo-site/EMBO%20Press%20Author%20Checklist-1642513524327.xlsx

Remove the figure from the main manuscript file and upload it as a separate high-resolution file. Figure legend and the table should be placed after references.

Please rename "Competing Interest Statement" to "Disclosure Statement & Competing Interests". We updated our journal's competing interests policy in January 2022 and request authors to consider both actual and perceived competing interests. Please review the policy https://www.embopress.org/competing-interests and update your competing interests if necessary.

In M&M, provide the antibody dilutions that were used for each antibody.

In the main text include a statement that informed consent was obtained from the patient and that the experiments conformed to the principles set out in the WMA Declaration of Helsinki and the Department of Health and Human Services Belmont Report.

Please remove "Author contributions" from the manuscript and specify author contributions in our submission system. CRediT has replaced the traditional author contributions section because it offers a systematic machine-readable author contributions format that allows for more effective research assessment. You are encouraged to use the free text boxes beneath each contributing author's name to add specific details on the author's contribution. More information is available in our guide to authors:
https://www.embopress.org/page/journal/17574684/authorguide#authorshipguidelines

We would welcome the submission of a revised version within three months for further consideration. Please let us know if you require longer to complete the revision.

I look forward to receiving your revised manuscript

Yours sincerely,

Zeljko Durdevic

Zeljko Durdevic
Editor
EMBO Molecular Medicine

*** Instructions to submit your revised manuscript ***

*** PLEASE NOTE *** As part of the EMBO Publications transparent editorial process initiative (see our Editorial at https://www.embopress.org/doi/pdf/10.1002/emmm.201000094), EMBO Molecular Medicine will publish online a Review Process File to accompany accepted manuscripts.

In the event of acceptance, this file will be published in conjunction with your paper and will include the anonymous referee reports, your point-by-point response and all pertinent correspondence relating to the manuscript. If you do NOT want this file to be published, please inform the editorial office at contact@embomolmed.org.

When submitting your revised manuscript, please include:

1) a .docx formatted version of the manuscript text (including Figure legends and tables)

2) Separate figure files*

3) supplemental information as Expanded View and/or Appendix. Please carefully check the authors guidelines for formatting Expanded view and Appendix figures and tables at https://www.embopress.org/page/journal/17574684/authorguide#expandedview

4) a letter INCLUDING the reviewer's reports and your detailed responses to their comments (as Word file).

5) The paper explained: EMBO Molecular Medicine articles are accompanied by a summary of the articles to emphasize the major findings in the paper and their medical implications for the non-specialist reader. Please provide a draft summary of your article highlighting
- the medical issue you are addressing,
- the results obtained and
- their clinical impact.
This may be edited to ensure that readers understand the significance and context of the research.
Please refer to any of our published articles for an example.

6) For more information: There is space at the end of each article to list relevant web links for further consultation by our readers. Could you identify some relevant ones and provide such information as well? Some examples are patient associations, relevant databases, OMIM/proteins/genes links, author's websites, etc...

7) Author contributions: the contribution of every author must be detailed in a separate section.

8) EMBO Molecular Medicine now requires a complete author checklist (https://www.embopress.org/page/journal/17574684/authorguide) to be submitted with all revised manuscripts. Please use the checklist as guideline for the sort of information we need WITHIN the manuscript. The checklist should only be filled with page numbers were the information can be found. This is particularly important for animal reporting, antibody dilutions (missing) and exact values and n that should be indicted instead of a range.

9) Every published paper now includes a 'Synopsis' to further enhance discoverability. Synopses are displayed on the journal webpage and are freely accessible to all readers. They include a short stand first (maximum of 300 characters, including space) as well as 2-5 one sentence bullet points that summarise the paper. Please write the bullet points to summarise the key NEW findings. They should be designed to be complementary to the abstract - i.e. not repeat the same text. We encourage inclusion of key acronyms and quantitative information (maximum of 30 words / bullet point). Please use the passive voice. Please attach these in a separate file or send them by email, we will incorporate them accordingly.

You are also welcome to suggest a striking image or visual abstract to illustrate your article. If you do please provide a jpeg file 550 px-wide x 400-px high.

10) A Conflict of Interest statement should be provided in the main text

11) Please note that we now mandate that all corresponding authors list an ORCID digital identifier. This takes <90 seconds to complete. We encourage all authors to supply an ORCID identifier, which will be linked to their name for unambiguous name identification.

Currently, our records indicate that the ORCID for your account is 0000-0001-9534-4102.

Please click the link below to modify this ORCID:
Link Not Available

12) The system will prompt you to fill in your funding and payment information. This will allow Wiley to send you a quote for the article processing charge (APC) in case of acceptance. This quote takes into account any reduction or fee waivers that you may be eligible for. Authors do not need to pay any fees before their manuscript is accepted and transferred to our publisher.

*Additional important information regarding Figures

Each figure should be given in a separate file and should have the following resolution:
Graphs 800-1,200 DPI
Photos 400-800 DPI
Colour (only CMYK) 300-400 DPI"

Figures are not edited by the production team. All lettering should be the same size and style; figure panels should be indicated by capital letters (A, B, C etc). Gridlines are not allowed except for log plots. Figures should be numbered in the order of their appearance in the text with Arabic numerals. Each Figure must have a separate legend and a caption is needed for each panel.

*Additional important information regarding figures and illustrations can be found at https://bit.ly/EMBOPressFigurePreparationGuideline. See also figure legend preparation guidelines: https://www.embopress.org/page/journal/17574684/authorguide#figureformat

The system will prompt you to fill in your funding and payment information. This will allow Wiley to send you a quote for the article processing charge (APC) in case of acceptance. This quote takes into account any reduction or fee waivers that you may be eligible for. Authors do not need to pay any fees before their manuscript is accepted and transferred to our publisher.

***** Reviewer's comments *****

Referee #1 (Remarks for Author):

In the manuscript, Etich and colleagues provided a number of clinical, genetic and cellular data supporting a role of TAPT1 (trans-membrane anterior-posterior transformation protein 1) as a causing gene of Osteogenesis imperfecta (OI). OI is a rare hereditary disorder of the connective tissue characterized by a reduced quality and/or quantity of bone matrix. This work was in line with a previous publication of this journal (PMID: 36652330), in which a TAPT1 intronic mutation has been identified and associated to dysregulation of collagen and extracellular matrix pathways in patients with a recessive OI and neonatal progeria syndrome.
This manuscript provides an further contribution regarding the role of TAPT1-modulated pathway in OI. I think that this story interesting for geneticists and researchers that work in the field of molecular diagnosis.

Specific points
1) Authors should include in the manuscript some data regarding the effect of the mutation (c.323T>G, p.Leu108Trp) on the function/structure/stability of the TAPT1 protein. Is this mutation located in any functional domain of the protein?
2) How could this mutation cause a reduction in protein expression or stability without affecting its mRNA level? The authors could for example test whether the mutated TAPT1 protein is unstable or mostly degraded by proteasome.
3) Line 95. These authors sustained: "To elucidate the underlying molecular pathomechanism, we used patient-derived fibroblasts and detected in vitro that the p.Leu108Trp variant of TAPT1 slightly reduced protein but not RNA levels (Fig 1C)". Analysis of the TAPT1 mRNA expression levels is in not present in Figure 1C (this is present in Figure EV1-A). Include this information in the text. In general figures of the EXTENDED VIEW MATERIAL are not cited in the text, it would be of help to the reader to include them in the text.
4) In the quantification of Fig1C (lower panel), P-value is not statistically significant (P-value = 0.06). And expression levels of TAPT1 in control fibroblasts are very different from each other.
5) What is the molecular mechanism by which the mutated version of TAPT1 induces SFRP1 upregulation? Is the WNT1 pathway differently activated in cells of the patient harboring the c.323T>G mutation? These authors should include in the text some information regarding an hypothetical molecular mechanism.
6) In addition include in the text more information regarding SFRP1 activity/function could be useful to the reader.
7) The explanation of the acronym "Secreted frizzled related protein 1 (SFRP1) must be included in the text at line 112.

We thank the referee for his/her valuable comments and suggestions which definitely helped to improve the manuscript. In the following point-to-point response we address each specific comment of the referee. Changes in the manuscript are highlighted (yellow).

Referee #1 (Remarks for Author):

In the manuscript, Etich and colleagues provided a number of clinical, genetic and cellular data supporting a role of TAPT1 (trans-membrane anterior-posterior transformation protein 1) as a causing gene of Osteogenesis imperfecta (OI). OI is a rare hereditary disorder of the connective tissue characterized by a reduced quality and/or quantity of bone matrix. This work was in line with a previous publication of this journal (PMID: 36652330), in which a TAPT1 intronic mutation has been identified and associated to dysregulation of collagen and extracellular matrix pathways in patients with a recessive OI and neonatal progeria syndrome.
This manuscript provides an further contribution regarding the role of TAPT1-modulated pathway in OI. I think that this story interesting for geneticists and researchers that work in the field of molecular diagnosis.

Specific points
1) Authors should include in the manuscript some data regarding the effect of the mutation (c.323T>G, p.Leu108Trp) on the function/structure/stability of the TAPT1 protein. Is this mutation located in any functional domain of the protein?

We thank the referee for the suggestion and now discuss the consequences of the newly identified *TAPT1* mutation for protein structure and stability. Visualization of the human TAPT1 protein structure with the mutation revealed that, beside others, the side-chains of Glu285, Asp306, Asp353, Lys356, His357 and Tyr371 are affected. Glu285, Asp306, Asp353, Lys356, His357 are located on different helices and are engaged in salt bridges, whereas His357 and Tyr371 are connected by a hydrogen bond. These long-range interactions between distant amino acids help to maintain the overall shape and stability of the protein. Thus, a central network of salt bridges and hydrogen bonds in the TAPT1 structure is most likely disrupted by the mutation (c.323T>G, p.Leu108Trp) resulting in a severe perturbation of the structural integrity.
In addition to simple visualization, we now performed a prediction of the mutation on protein stability employing the multiple Cutoff Scanning Matrix method (mCSM; https://biosig.lab.uq.edu.au/mcsm/) and the predicted structure of TAPT1. This analysis predicted an estimated loss of 2.1 kcal/mol in energy and the mutation is classified as ‘highly destabilizing’. Destabilized proteins are likely to be degraded, which would explain the reduced amount of protein that is detected in the patient, while the transcript levels are normal (see also point 2 of the reviewer).

In the revised manuscript, we now discuss the perturbation of salt bridges and hydrogens bonds by the exchange of leucine to tryptophane at position 108 and include the information on mutant protein destabilization (page 3, lines 68-75). In addition, we highlight the involvement of these amino acids in the figure legend of Fig 1B (page 9, line 241-243) and include a paragraph on the bioinformatic analysis methods for mutant protein structure and stability in the Appendix (Appendix page 9, line 160-167). We cannot speculate on the impact of the mutation on TAPT1 functional domains as a precise molecular function has yet to be ascertained for TAPT1.

2) How could this mutation cause a reduction in protein expression or stability without affecting its mRNA level? The authors could for example test whether the mutated TAPT1 protein is unstable or mostly degraded by proteasome.

We appreciate the suggestion of the referee and experimentally addressed the question how the identified mutation could cause a reduction in protein levels without affecting mRNA levels. There are two key cellular mechanisms in eukaryotes that mediate protein turnover and remove misfolded or dysfunctional proteins, the ubiquitin-proteasome pathway and the lysosomal proteolysis pathway. Using the potent and selective, cell permeable, peptidyl-aldehyde type proteasome inhibitor MG132, we could demonstrate that inhibition of the proteasomal degradation machinery restored TAPT1 protein levels to the levels of control cells. In contrast, TAPT1 levels remained reduced in patient cells when lysosomal degradation was inhibited by Bafilomycin A1, which acts as a highly potent and specific inhibitor of vacuolar-type ATPase to block the fusion of autophagosomes with lysosomes. These results indicate that a presumably misfolded and destabilized mutant TAPT1 protein is mainly degraded by the proteasome pathway. The new experimental data are now included in Appendix Fig 1B (Appendix page 2, line 38-43) and described in the revised manuscript (page 3, lines 81-86).

3) Line 95. These authors sustained: "To elucidate the underlying molecular pathomechanism, we used patient-derived fibroblasts and detected in vitro that the p.Leu108Trp variant of TAPT1 slightly reduced protein but not RNA levels (Fig 1C)". Analysis of the TAPT1 mRNA expression levels is in not present in Figure 1C (this is present in Figure EV1-A). Include this information in the text. In general figures of the EXTENDED VIEW MATERIAL are not cited in the text, it would be of help to the reader to include them in the text.

We agree with the referee that it would help to cite the EXTENDED VIEW MATERIAL in the text. However, we did not include the information on the additional figures in the initially submitted manuscript due to editorial restrictions of the journal for Correspondence articles. As we felt that the additional figures and detailed material and methods are important for the study, we left this to the editor’s discretion. We now follow his recommendation and make the Expanded View file available as "Appendix" with a table of content. The figures are renamed to "Appendix Figure S1" etc. and are now described and cited in the main text (page 3, line 80-93; page 4, line 101, 102, 106).

4) In the quantification of Fig1C (lower panel), P-value is not statistically significant (P-value = 0.06). And expression levels of TAPT1 in control fibroblasts are very different from each other.

We thank the referee for the comment. The use of several primary control cells definitely revealed a clear variability in the expression of TAPT1. We also acknowledge that the availability of only one patient with the identified mutation is a limitation of our study. Therefore, we believe that the use of multiple primary control cells that show biological variability will minimize over-interpretation of data due to individual characteristics of the donors. We included a statement about biological variability of control cell populations in the Appendix (Appendix page 9, line 174-176).

5) What is the molecular mechanism by which the mutated version of TAPT1 induces SFRP1 upregulation? Is the WNT1 pathway differently activated in cells of the patient harboring the c.323T>G mutation? These authors should include in the text some information regarding an hypothetical molecular mechanism.

We appreciate the suggestion of the referee to include some thoughts regarding a hypothetical molecular mechanism. We now speculate that TAPT1 protein is involved in the transcriptional regulation of *SFRP1* gene expression. A number of cilia-associated proteins are also persistent in the nucleus and potentially play a role there. Among these myocardin-related transcription factor and serum response factor act not only as transcription factors, but also as constituents of primary cilia impacting on ciliary protein-protein interactions (Speight *et al*, 2021). It is therefore conceivable that TAPT1 at the centrosome/ciliary base could interact with transcription-modulating proteins and regulate their nuclear translocation thereby inhibiting downstream *SFRP1* expression. Thus, we speculate that mutant TAPT1 protein, due to reduced protein stability and levels as well as diminished protein interactions, lacks the ability to regulate *SFRP1* silencing and, consequently, *SFRP1* gene expression is induced impairing bone remodeling. We include this hypothetical molecular mechanism, supported by additional references, in the revised manuscript (page 4-5, line 122-132).
Unfortunately, we do not have access to patient osteoblasts to carefully address changes in WNT signal transduction. This will be needed in future experiments to unequivocally determine the involvement of WNT pathway in the patient. Nevertheless, the bone features are strikingly similar to OI type XV, in which WNT signaling is inhibited by WNT1 mutation. Therefore, the bone phenotype in our patient might be at least in part caused by deregulated WNT signaling.

6) In addition include in the text more information regarding SFRP1 activity/function could be useful to the reader.

We are grateful for this comment. SFRP1 is a secreted glycoprotein that can antagonize WNT activity by directly binding to the WNT ligands through its amino-terminal cysteine-rich domain and eventually can

inhibit the interaction of the WNT ligand with the Frizzled receptor. Thereby, β-catenin-mediated gene transcription is prevented, the pathway inhibited and bone formation reduced. The importance of this regulation was previously demonstrated by showing that the deletion of *SFRP1* induced activation of canonical WNT signaling in bone, up-regulation of osteoblast proliferation/differentiation and increase in trabecular bone formation in aged mice (Bodine *et al*, 2004). More importantly, therapeutic inhibition of SFRP1 in a murine calvarial organ culture assay and ovariectomy-induced osteoporotic mice led to increased anabolic activity, demonstrating the importance of SFRP1 as a potential therapeutic target to increase bone formation (Bodine *et al*, 2009; García-García *et al*, 2022). We expanded the paragraph on the mode of action of SFRP1 as a well-established antagonist of the WNT signaling pathway in the revised manuscript and included relevant literature (page 4, line 109-120).

7) The explanation of the acronym "Secreted frizzled related protein 1 (SFRP1) must be included in the text at line 112.

We thank the reviewer for this comment and included the explanation of the acronym when mentioning SFRP1 for the first time (page 4, line 106-107).

REFERENCES

Bodine PVN, Stauffer B, Ponce-de-Leon H, Bhat RA, Mangine A, Seestaller-Wehr LM, Moran RA, Billiard J, Fukayama S, Komm BS, *et al* (2009) A small molecule inhibitor of the Wnt antagonist secreted frizzled-related protein-1 stimulates bone formation. *Bone* 44: 1063–1068

Bodine PVN, Zhao W, Kharode YP, Bex FJ, Lambert AJ, Goad MB, Gaur T, Stein GS, Lian JB & Komm BS (2004) The Wnt antagonist secreted frizzled-related protein-1 is a negative regulator of trabecular bone formation in adult mice. *Mol Endocrinol* 18: 1222–1237

García-García P, Reyes R, García-Sánchez D, Pérez-Campo FM, Rodríguez-Rey JC, Évora C, Díaz-Rodríguez P & Delgado A (2022) Nanoparticle-mediated selective Sfrp-1 silencing enhances bone density in osteoporotic mice. *J Nanobiotechnology* 20: 1–19

Speight P, Rozycki M, Venugopal S, Szászi K, Kofler M & Kapus A (2021) Myocardin-related transcription factor and serum response factor regulate cilium turnover by both transcriptional and local mechanisms. *iScience* 24: 102739

Further changes were made according to the Editor's comments in the decision letter and are highlighted (yellow).

1) The figure was removed from the main manuscript and uploaded as a separate high-resolution file.
2) Disclosure Statement & Competing Interests were updated (page 7, line 180-181).

3) Antibody dilutions were provided in M&M (Appendix page 11-12, line 228, 238, 242, 250-252).

4) A statement that informed consent was obtained from the patient and that the experiments conformed to the principles set out in the WMA Declaration of Helsinki and the Department of Health and Human Services Belmont Report was included in the main manuscript (page 3, line 77-79) and the Appendix (Appendix page 7, line 122-124).

5) Author contributions were removed from the manuscript and are specified in the submission system.

6) Source data files were provided. To comply with source data requirements and after consultation with the SourceData Scientific Coordinator Hannah Sonntag, the inserts/close ups of Fig 1F were replaced.

25th May 2023

Dear Dr. Rehberg,

We are pleased to inform you that your manuscript is accepted for publication and is now being sent to our publisher to be included in the next available issue of EMBO Molecular Medicine.

Please read below for additional IMPORTANT information regarding your article, its publication and the production process.

Congratulations on your interesting work,

Zeljko Durdevic

Zeljko Durdevic
Editor
EMBO Molecular Medicine

Follow us on Twitter @EmboMolMed
Sign up for eTOCs at embopress.org/alertsfeeds

*** *** *** IMPORTANT INFORMATION *** *** ***

SPEED OF PUBLICATION
The journal aims for rapid publication of papers, using using the advance online publication "Early View" to expedite the process: A properly copy-edited and formatted version will be published as "Early View" after the proofs have been corrected. Please help the Editors and publisher avoid delays by providing e-mail address(es), telephone and fax numbers at which author(s) can be contacted.

Should you be planning a Press Release on your article, please get in contact with embomolmed@wiley.com as early as possible, in order to coordinate publication and release dates.

LICENSE AND PAYMENT:

All articles published in EMBO Molecular Medicine are fully open access: immediately and freely available to read, download and share.

EMBO Molecular Medicine charges an article processing charge (APC) to cover the publication costs. You, as the corresponding author for this manuscript, should have already received a quote with the article processing fee separately. Please let us know in case this quote has not been received.

Once your article is at Wiley for editorial production you will receive an email from Wiley's Author Services system, which will ask you to log in and will present you with the publication license form for completion. Within the same system the publication fee can be paid by credit card, an invoice, pro forma invoice or purchase order can be requested.

Payment of the publication charge and the signed Open Access Agreement form must be received before the article can be published online.

PROOFS

You will receive the proofs by e-mail approximately 2 weeks after all relevant files have been sent o our Production Office. Please return them within 48 hours and if there should be any problems, please contact the production office at embopressproduction@wiley.com.

Please inform us if there is likely to be any difficulty in reaching you at the above address at that time. Failure to meet our deadlines may result in a delay of publication.

All further communications concerning your paper proofs should quote reference number EMM-2023-17528-V2 and be directed to the production office at embopressproduction@wiley.com.

Thank you,

Zeljko Durdevic
Editor
EMBO Molecular Medicine

EMBO Press Author Checklist

Corresponding Author Name: Julia Etich and Mirko Rehberg	
Journal Submitted to: EMBO Molecular Medicine	
Manuscript Number: EMM-2023-17528	

USEFUL LINKS FOR COMPLETING THIS FORM
The EMBO Journal - Author Guidelines
EMBO Reports - Author Guidelines
Molecular Systems Biology - Author Guidelines
EMBO Molecular Medicine - Author Guidelines

Reporting Checklist for Life Science Articles (updated January
This checklist is adapted from Materials Design Analysis Reporting (MDAR) Checklist for Authors. MDAR establishes a minimum set of requirements in transparent reporting in the life sciences (see Statement of Task: 10.31222/osf.io/9sm4x). Please follow the journal's guidelines in preparing your
Please note that a copy of this checklist will be published alongside your article.

Abridged guidelines for figures
1. Data
The data shown in figures should satisfy the following conditions:
- the data were obtained and processed according to the field's best practice and are presented to reflect the results of the experiments in an accurate and unbiased manner.
- ideally, figure panels should include only measurements that are directly comparable to each other and obtained with the same assay.
- plots include clearly labeled error bars for independent experiments and sample sizes. Unless justified, error bars should not be shown for technical
- if n<5, the individual data points from each experiment should be plotted. Any statistical test employed should be justified.
- Source Data should be included to report the data underlying figures according to the guidelines set out in the authorship guidelines on Data

2. Captions
Each figure caption should contain the following information, for each panel where they are relevant:
- a specification of the experimental system investigated (eg cell line, species name).
- the assay(s) and method(s) used to carry out the reported observations and measurements.
- an explicit mention of the biological and chemical entity(ies) that are being measured.
- an explicit mention of the biological and chemical entity(ies) that are altered/varied/perturbed in a controlled manner.
- the exact sample size (n) for each experimental group/condition, given as a number, not a range;
- a description of the sample collection allowing the reader to understand whether the samples represent technical or biological replicates (including how many animals, litters, cultures, etc.).
- a statement of how many times the experiment shown was independently replicated in the laboratory.
- definitions of statistical methods and measures:
  - common tests, such as t-test (please specify whether paired vs. unpaired), simple χ2 tests, Wilcoxon and Mann-Whitney tests, can be unambiguously identified by name only, but more complex techniques should be described in the methods section;
  - are tests one-sided or two-sided?
  - are there adjustments for multiple comparisons?
  - exact statistical test results, e.g., P values = x but not P values < x;
  - definition of 'center values' as median or average;
  - definition of error bars as s.d. or s.e.m.

Please complete ALL of the questions below.
Select "Not Applicable" only when the requested information is not relevant for your study.

Materials

Newly Created Materials	Information included in the manuscript?	In which section is the information available? (Reagents and Tools Table, Materials and Methods, Figures, Data Availability Section)
New materials and reagents need to be available; do any restrictions apply?	Not Applicable	

Antibodies	Information included in the manuscript?	In which section is the information available? (Reagents and Tools Table, Materials and Methods, Figures, Data Availability Section)
For **antibodies** provide the following information:<br>- Commercial antibodies: RRID (if possible) or supplier name, catalogue number and or/clone number<br>- Non-commercial: RRID or citation	Yes	Appendix Material and Methods

DNA and RNA sequences	Information included in the manuscript?	In which section is the information available? (Reagents and Tools Table, Materials and Methods, Figures, Data Availability Section)
**Short novel DNA or RNA including primers, probes**: provide the sequences.	Yes	Appendix Material and Methods

Cell materials	Information included in the manuscript?	In which section is the information available? (Reagents and Tools Table, Materials and Methods, Figures, Data Availability Section)
**Cell lines:** Provide species information, strain. Provide accession number in repository **OR** supplier name, catalog number, clone number, and/**OR** RRID.	Not Applicable	
**Primary cultures:** Provide species, strain, sex of origin, genetic modification status.	Yes	Appendix Material and Methods
Report if the cell lines were recently **authenticated** (e.g., by STR profiling) and tested for mycoplasma contamination.	Not Applicable	

Experimental animals	Information included in the manuscript?	In which section is the information available? (Reagents and Tools Table, Materials and Methods, Figures, Data Availability Section)
**Laboratory animals or Model organisms:** Provide species, strain, sex, age, genetic modification status. Provide accession number in repository **OR** supplier name, catalog number, clone number, **OR** RRID.	Not Applicable	
**Animal observed in or captured from the field:** Provide species, sex, and age where possible.	Not Applicable	
Please detail **housing and husbandry conditions**.	Not Applicable	

Plants and microbes	Information included in the manuscript?	In which section is the information available? (Reagents and Tools Table, Materials and Methods, Figures, Data Availability Section)
**Plants:** provide species and strain, ecotype and cultivar where relevant, unique accession number if available, and source (including location for collected wild specimens).	Not Applicable	
**Microbes:** provide species and strain, unique accession number if available, and source.	Not Applicable	

Human research participants	Information included in the manuscript?	In which section is the information available? (Reagents and Tools Table, Materials and Methods, Figures, Data Availability Section)
If collected and within the bounds of privacy constraints report on age, sex and gender or ethnicity for all study participants.	Yes	Main text, Appendix Material and Methods

Core facilities	Information included in the manuscript?	In which section is the information available? (Reagents and Tools Table, Materials and Methods, Figures, Data Availability Section)
If your work benefited from core facilities, was their service mentioned in the acknowledgments section?	Yes	Acknowledgements

Design

Study protocol	Information included in the manuscript?	In which section is the information available? (Reagents and Tools Table, Materials and Methods, Figures, Data Availability Section)
If study protocol has been **pre-registered, provide DOI in the manuscript**. For clinical trials, provide the trial registration number **OR** cite DOI.	Not Applicable	
Report the **clinical trial registration number** (at ClinicalTrials.gov or equivalent), where applicable.	Not Applicable	

Laboratory protocol	Information included in the manuscript?	In which section is the information available? (Reagents and Tools Table, Materials and Methods, Figures, Data Availability Section)
Provide DOI OR other citation details if **external detailed step-by-step protocols** are available.	Yes	Appendix Material and Methods

Experimental study design and statistics	Information included in the manuscript?	In which section is the information available? (Reagents and Tools Table, Materials and Methods, Figures, Data Availability Section)
Include a statement about **sample size** estimate even if no statistical methods were used.	Yes	Figure legend
Were any steps taken to minimize the effects of subjective bias when allocating animals/samples to treatment (e.g. **randomization procedure**)? If yes, have they been described?	Not Applicable	
Include a statement about **blinding** even if no blinding was done.	Yes	Appendix Material and Methods
Describe **inclusion/exclusion criteria** if samples or animals were excluded from the analysis. Were the criteria pre-established?<br><br>If sample or data points were omitted from analysis, report if this was due to attrition or intentional exclusion and provide justification.	Yes	Appendix Material and Methods
For every figure, are **statistical tests** justified as appropriate? Do the data meet the assumptions of the tests (e.g., normal distribution)? Describe any methods used to assess it. Is there an estimate of variation within each group of data? Is the variance similar between the groups that are being statistically compared?	Yes	Appendix Material and Methods

Sample definition and in-laboratory replication	Information included in the manuscript?	In which section is the information available? (Reagents and Tools Table, Materials and Methods, Figures, Data Availability Section)
In the figure legends: state number of times the experiment was **replicated** in laboratory.	Yes	Figure legend
In the figure legends: define whether data describe **technical or biological replicates**.	Yes	Figure legend

Ethics

Ethics	Information included in the manuscript?	In which section is the information available? (Reagents and Tools Table, Materials and Methods, Figures, Data Availability Section)
Studies involving **human participants**: State details of **authority granting ethics approval** (IRB or equivalent committee(s), provide reference number for approval.	Yes	Main text, Appendix Material and Methods
Studies involving **human participants**: Include a statement confirming that **informed consent** was obtained from all subjects and that the experiments conformed to the principles set out in the WMA Declaration of Helsinki and the Department of Health and Human Services Belmont Report.	Yes	Main text, Appendix Material and Methods
Studies involving **human participants: For** publication of **patient photos**, include a statement confirming that consent to publish was obtained.	Yes	Appendix Material and Methods
Studies involving experimental **animals**: State details of **authority granting ethics approval** (IRB or equivalent committee(s), provide reference number for approval. Include a statement of compliance with ethical regulations.	Not Applicable	
Studies involving **specimen and field samples:** State if relevant **permits** obtained, provide details of authority approving study; if none were required, explain why.	Not Applicable	

Dual Use Research of Concern (DURC)	Information included in the manuscript?	In which section is the information available? (Reagents and Tools Table, Materials and Methods, Figures, Data Availability Section)
Could your study fall under dual use research restrictions? Please check biosecurity documents and list of **select agents and toxins** (CDC): https://www.selectagents.gov/sat/list.htm	Not Applicable	
If you used a select agent, is the security level of the lab appropriate and reported in the manuscript?	Not Applicable	
If a study is subject to dual use research of concern regulations, is the name of the **authority granting approval and reference number** for the regulatory approval provided in the manuscript?	Not Applicable	

Reporting

The MDAR framework recommends adoption of discipline-specific guidelines, established and endorsed through community initiatives. Journals have their own policy about requiring specific guidelines and recommendations to complement MDAR.

Adherence to community standards	Information included in the manuscript?	In which section is the information available? (Reagents and Tools Table, Materials and Methods, Figures, Data Availability Section)
State if relevant guidelines or checklists (e.g., **ICMJE, MIBBI, ARRIVE, PRISMA**) have been followed or provided.	Not Applicable	
For **tumor marker prognostic studies,** we recommend that you follow the **REMARK** reporting guidelines (see link list at top right). See author guidelines, under 'Reporting Guidelines'. Please confirm you have followed these guidelines.	Not Applicable	
For **phase II and III randomized controlled trials**, please refer to the **CONSORT** flow diagram (see link list at top right) and submit the CONSORT checklist (see link list at top right) with your submission. See author guidelines, under 'Reporting Guidelines'. Please confirm you have submitted this list.	Not Applicable	

Data Availability

Data availability	Information included in the manuscript?	In which section is the information available? (Reagents and Tools Table, Materials and Methods, Figures, Data Availability Section)
Have **primary datasets** been deposited according to the journal's guidelines (see 'Data Deposition' section) and the respective accession numbers provided in the Data Availability Section?	Not Applicable	
Were **human clinical and genomic datasets** deposited in a public access-controlled repository in accordance to ethical obligations to the patients and to the applicable consent agreement?	Yes	Appendix Material and Methods
Are **computational models** that are central and integral to a study available without restrictions in a machine-readable form? Were the relevant accession numbers or links provided?	Yes	Appendix Material and Methods
If publicly available data were reused, provide the respective **data citations in the reference list**.	Not Applicable	
